# ANXA1 promotes intrahepatic cholangiocarcinoma proliferation and growth by regulating glutamine metabolism through GOT1 stabilization

**DOI:** 10.1186/s13046-025-03400-z

**Published:** 2025-05-19

**Authors:** Yanyu Gong, Liwen Chen, Hao Wang, Dijie Zheng, Futang Li, Changhao Wu, Yongning Li, Yazhu Deng, Zhiwei He, Chao Yu

**Affiliations:** 1 Department of Hepatobiliary Surgery, The Affiliated Hospital of Guizhou Medical University, Guizhou Medical University, Guiyang, 550001 China; 2 School of Clinical Medicine, Guizhou Medical University, Guiyang, 550004 China; 3 Guizhou Provincial Institute of Hepatobiliary, Pancreatic and Splenic Diseases, Guiyang, 550001 China; 4 Key Laboratory of Liver, Pancreas and Spleen of Guizhou Medical University, Gallbladder, Guiyang, 550001 China; 5 Guizhou Provincial Clinical Medical Research Center of Hepatobiliary Surgery, Guiyang, 550004 Guizhou China; 6 Key Laboratory of Hepatobiliary and Pancreatic Diseases Treatment and Bioinformatics Research, Guizhou Medical University, Guiyang, 550001 China; 7 Department of Hepatobiliary Surgery, Shenzhen University General Hospital, Shenzhen, 518052 China

**Keywords:** ICC, ANXA1, GOT1, Glutamine metabolism, Oxidative stress

## Abstract

**Background:**

Intrahepatic cholangiocarcinoma (ICC) is a malignant tumor with a poor prognosis, marked by a postoperative recurrence rate of 50–60% and a 5-year survival rate of 8–30%. Abnormal tumor metabolism, particularly, amino acid metabolism, plays a key role in malignant progression. However, the molecular mechanisms linking amino acid metabolism to ICC progression remain unclear.

**Methods:**

Bioinformatics was used to identity the key amino acid metabolism related gene in ICC, qRT-PCR, western blotting and immunohistochemical (IHC) were used to detect the expression of ANXA1 in normal tissues or ICC tissues and cells at mRNA and protein levels. The effects of ANXA1 on the proliferation ability of ICC in vitro and in vivo were investigated using CCK8, cloning formation experiment, EdU, IHC, nude mice subcutaneous tumorigenesis model. Immunoprecipitation, mass spectrometry analysis, protein ubiquitin level detection test, immunofluorescence co-localization, and redox stress metabolite detection test were used to explore the metabolism-related regulatory mechanism of ANXA1.

**Results:**

we employed bioinformatics analysis to classify ICC into metabolic subgroups with distinct prognoses and identified the associated biomarker Annexin A1(ANXA1), whose high expression is correlated with poor prognosis and promotes ICC development. Mass spectrometry analysis revealed that ANXA1 interacts with the key enzyme in glutamine metabolism, glutamic-oxaloacetic transaminase 1(GOT1). Through in vitro and in vivo experiments, overexpressed ANXA1 stabilizes GOT1 by recruiting the deubiquitinase USP5. This stabilization enhances glutamine uptake, as well as the production of aspartate and glutamate, which in turn reduces oxidative stress, thereby promoting tumor cell growth. Moreover, knockdown of ANXA1 combined with glutamine uptake inhibition significantly suppressed ICC cell proliferation and Inhibited subcutaneous tumor formation and growth.

**Conclusions:**

These results suggest that the ANXA1/USP5/GOT1 axis promotes glutamine metabolism and ICC proliferation and growth. Inhibiting ANXA1 alongside glutamine uptake inhibition offers a promising strategy for treating ICC.

**Supplementary Information:**

The online version contains supplementary material available at 10.1186/s13046-025-03400-z.

## Introduction

Intrahepatic cholangiocarcinoma (ICC) is a nearly universally fatal malignancy and represents the second most common primary liver cancer, accounting for 20% of liver malignancies and 3% of all gastrointestinal malignancies [1–3]. Unlike extrahepatic cholangiocarcinoma, ICC rarely manifests with typical symptoms of biliary obstruction, such as obstructive jaundice, particularly in its early stages [4]. Even after curative surgical resection, the 5-year overall survival (OS) rate remains dismal, ranging from 20 to 35% [5]. Advances in surgical techniques and adjuvant chemotherapy have not significantly reduced the high rates of local recurrence following complete surgical resection. Therefore, therapeutic strategies beyond surgery and chemotherapy are urgently needed [6, 7].

Research has shown that ICC onset and progression are closely related to the dysregulation of metabolic pathways [8]. Although targeting metabolic reprogramming shows promise, it is still in its nascent stages as a treatment strategy [9]. Abnormal metabolism is a key characteristic of tumors and is closely associated with various aspects of tumor occurrence, progression, metastasis, and response to treatment [10]. As research cell metabolism continues to deepen, it has been discovered that apart from glucose metabolism dysregulation, other nutrients within tumor cells also undergo abnormal metabolic changes glutamine metabolism [11]. Glutamine metabolism is essential for the biosynthesis of tumor cell precursors, maintaining redox homeostasis and providing energy, thereby promoting cell proliferation, migration, and invasion [12–14]. Glutamic acid, a product of glutamine metabolism, is one of the raw materials for the synthesis of glutathione (GSH), a representative of the reducing agent, and can neutralize peroxides to protect tumor cells from oxidative stress. Tumor cells enhance their antioxidant capacity through glutamine metabolism to maintain their continued survival [15, 16].

Glutamate-oxaloacetate transaminase 1 (GOT1) is a critical enzyme involved in amino acid metabolism and cell proliferation in various malignancies [17]. GOT1 expression is upregulated in cancers such as pancreatic ductal adenocarcinoma, colorectal cancer, breast cancer, prostate cancer, acute myeloid leukemia, and hepatocellular carcinoma [18, 19]. Daniel et al. demonstrated that targeting GOT1 induces pancreatic cancer cell death, highlighting its potential as a therapeutic target [20]. Regulation of GOT1 stability and activity is poorly understood, but deubiquitinases, which play pivotal roles in maintaining the stability of oncogenes, are likely contributors [21]. Among these, ubiquitin-specific protease 5 (USP5) has emerged as a key regulator of cellular functions through its ability to remove ubiquitin chains from target proteins [22–24]. USP5 is implicated in the progression of multiple cancer types [25]. However, the interactions between GOT1 and other binding partners, particularly USP5, remain unexplored.

Annexin A1 (ANXA1), a member of the annexin family, is a multifunctional protein involved in cellular signal transduction, inflammation regulation, and apoptosis. Within cells, ANXA1 functions as a scaffold protein, playing a critical role in cell signaling and membrane dynamics [26]. Other annexin family members, such as ANXA2 and ANXA6, have been shown to regulate endocytosis. For instance, ANXA6 acts as a scaffold protein for protein kinase C (PKC) α and p120 GTPase-activating protein (GAP), negatively regulating the EGFR/Ras/MAPK pathway and promoting lysosomal degradation of EGFR [27, 28]. ANXA1 has also been implicated in tumor metabolism, including lipid metabolism and cholesterol metabolism, by modulating metabolic enzyme activity and influencing the progression of both malignant and benign diseases [29–31]. However, the role of ANXA1 in the carcinogenesis and progression of ICC remains unclear.

In this study, we identified ANXA1 as a novel scaffold protein facilitating the interaction between USP5 and GOT1, promoting deubiquitination and stabilizing GOT1 and further regulating glutamine metabolism. ANXA1 is highly expressed in patients with ICC and promotes its development and progression. Further mass spectrometry (MS) analysis identified GOT1 and USP5 as binding partners of ANXA1. Mechanistically, ANXA1 promotes the binding of USP5 to GOT1, thus preventing the degradation of GOT1 via the ubiquitin-proteasome pathway and promoting glutamine metabolism to enhance the oxidative stress adaptation of tumor cells and promote tumor growth. More importantly, we determined the impact of ANXA1 on amino acid metabolism in ICC and gained a deeper understanding of the pathogenic mechanisms in patients with ICC, thereby identifying potential therapeutic targets for ICC.

## Materials and methods

### Tissue samples

A total of 78 patients’ samples diagnosed with intrahepatic cholangiocarcinoma (ICC) were collected from the Affiliated Hospital of Guizhou Medical University between September 2017 and September 2022. All patients underwent radical surgery and were diagnosed with ICC based on preoperative imaging examinations and postoperative pathology and had not received any anti-tumor treatment before surgery. Detailed clinical information regarding these samples is provided in Supplementary Table 1, while the clinical baseline characteristics are summarized in Table 1 of Fig. 2. Immediately following excision, the tissues were either frozen in liquid nitrogen or fixed using 4% paraformaldehyde. Each specimen underwent histological and pathological examination, grading, and staging conducted by two experienced pathologists, adhering to the AJCC 8th edition criteria. This study adhered to ethical standards and was approved by the Ethics Committee of the First Affiliated Hospital of Guizhou Medical University, with informed consent obtained for all tissue samples.

**Fig. 1:**
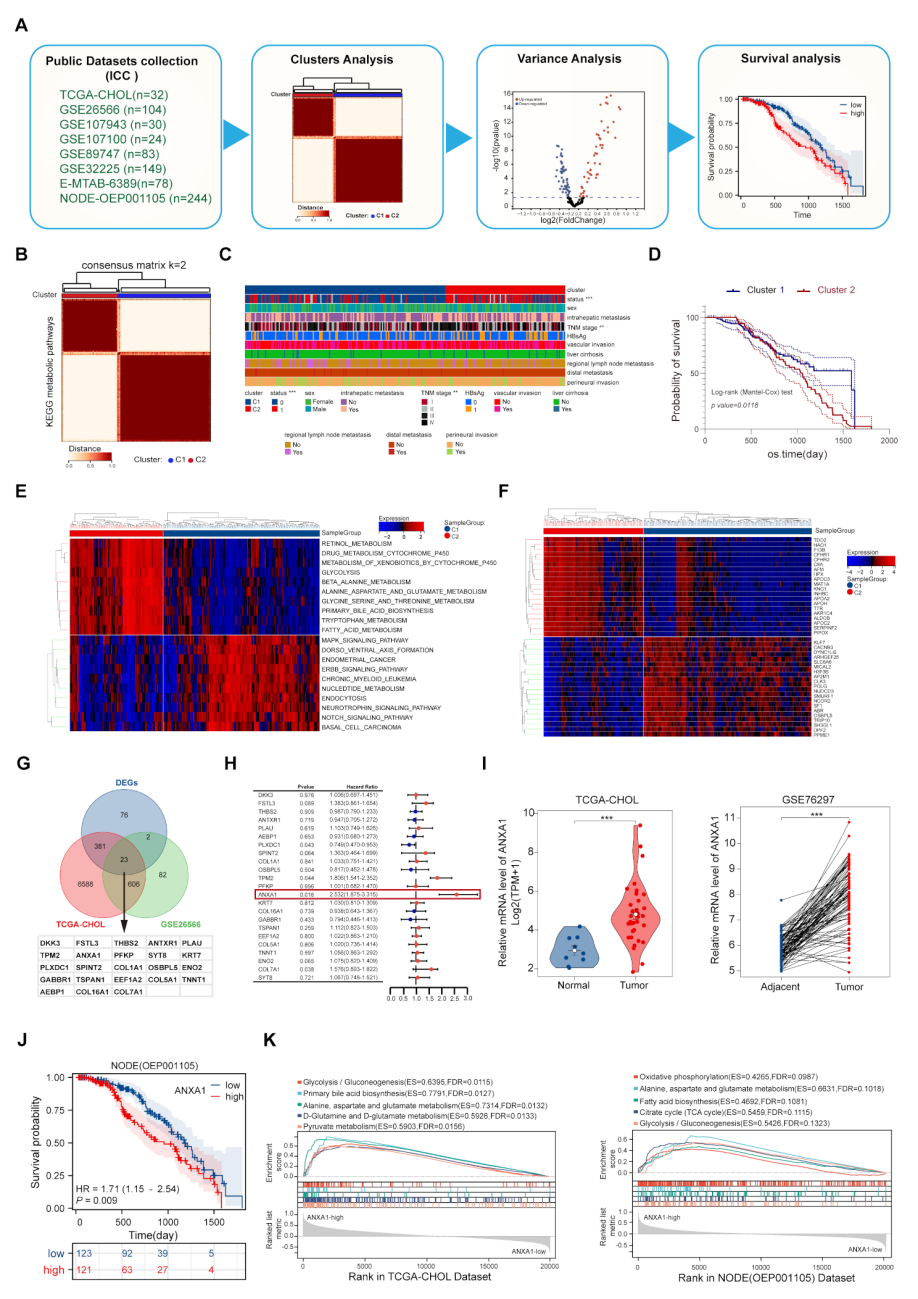
Identification of genes highly associated with metabolism and prognosis of ICC using bioinformatics. **A**: Workflow for the screening process. **B**: Consensus clustering analysis based on 76 KEGG metabolic pathways from 744 ICC RNA-seq. **C**: Heatmap of clinical characteristics with survival information. **D**: Survival difference between the two metabolic clusters (Log-rank test). **E**: Top 10 differential metabolic pathways between the two clusters. **F**: Top 20 differential genes between the two metabolic clusters. **G**: Intersection of differential metabolic genes with TCGA-CHOL and GSE26566. **H**: Univariate COX regression analysis of 23 metabolic differential genes. **I**: Expression levels of ANXA1 in TCGA-CHOL (Mann–Whitney U test, ***, *p* < 0.001) and GSE76297(Wilcoxon matched-pairs test, ***, *p* < 0.001). **J**: Kaplan-Meier analysis of overall survival in low and high ANXA1 expression groups in dataset OEP00001105(Log-rank test). **K**: Top 5 metabolic pathways from the single-gene GSEA enrichment analysis of ANXA1 in the TCGA-CHOL and NODE (OEP001105) datasets

### Bioinformatics analysis

#### The integration, clustering, and functional analysis of transcriptome data from samples

In order to find genes that play a role in the occurrence, development and metabolism of ICC, we used the following steps to screen candidate genes: ①Data acquisition: Gene expression and related clinical information about ICC patients were obtained from The Cancer Genome Atlas (TCGA, *n* = 32), Gene Expression Omnibus (GEO) database (GSE26566 (*n* = 104), GSE107943 (*n* = 30), GSE107100 (*n* = 24), GSE89747 (*n* = 83), GSE32225 (*n* = 149), China National Omics Data Encyclopedia NODE (OEP001105, *n* = 244), European Bioinformatics Institute E-MTAB-6389 (*n* = 78), a total of 744 samples and 76 KEGG metabolic related genes in 12 categories(Download it from https://www.genome.jp/kegg/pathway.html#metabolism); To integrate transcriptomic sequencing data from multiple platforms and centers and eliminate systematic biases caused by differences in sample origins, sequencing platforms, or experimental batches, we performed data integration and batch effect correction using R software. First, gene annotation was standardized across datasets, and commonly expressed genes were identified to construct a merged expression matrix. Expression values were then log2-transformed [log2(x + 1)] to minimize the influence of extreme values on subsequent analyses. Batch variables were defined based on sample origin information, and the ComBat algorithm from the R package “sva, version: 3.55.0 " was employed to remove batch effects. ComBat utilizes an empirical Bayesian method to effectively eliminate non-biological variations while preserving true biological differences. Principal component analysis (PCA) was performed to visualize and evaluate the effectiveness of batch correction. The batch-corrected expression matrix was then used for downstream analyses, including differential gene expression, pathway enrichment, and molecular subtype analyses. Gene sets of pathways related to each ICC sample were analyzed, and the metabolic scores of each ICC sample were calculated using GSVA analysis (GSVA package: version 2.01) based on the R tool (version: R-4.2.2); ②Based on the metabolic score of each sample, the R package “ConsensusClusterPlus, version: 1.70.0” was used to perform consistency clustering analysis to find the optimal number of clusters; ③Metabolic grouping was performed based on the optimal number of clusters and their prognostic differences were analyzed, and the “limma, version: 3.16 " package was used to identify differentially expressed metabolic pathways and genes: differentially expressed genes were used for subsequent GO or KEGG enrichment analysis; ④Survival analysis of ICC candidate genes: survival information was extracted from ICC patients, and then survival analysis was performed using the R packages “Survival, version: 3.3-1” and “survminer, version:0.4.9”. The metabolic gene set used in this study was from the “KEGG PATHWAY Database”. Genes identified after using *P* < 0.05 and FDR < 0.25 were considered significant or statistically significant.⑤For the single-gene GSEA analysis, we obtained the GSEA software (version 3.0) from the GSEA website (http://software.broadinstitute.org/gsea/index.jsp). Based on the expression level of ANXA1, the samples were divided into a high expression group (≥ 50%) and a low expression group (< 50%). We downloaded the relevant gene sets “c2.cp.kegg.v7.4.symbols.gmt” subset from The Molecular Signatures Database(MSigDB) (http://www.gsea-msigdb.org/gsea/downloads.jsp) to evaluate related pathways and molecular mechanisms. Using gene expression profiles and phenotype grouping, the minimum gene set was set to 5 and the maximum gene set to 5000, with 1,000 resampling. A p-value of < 0.05 and an FDR of < 0.25 were considered statistically significant.

#### Prediction of ubiquitination sites on GOT1

To preliminarily explore the potential ubiquitination sites of GOT1, we utilized a ubiquitination site prediction tool based on MATLAB Compiler Runtime (MCR, version: R2009a MCR 7.10). The FASTA sequence of the human GOT1 protein was obtained from the Uniport database and submitted to the prediction system. This tool employs a support vector machine (SVM)-based classification model that distinguishes ubiquitinated from non-ubiquitinated lysine (K) residues using machine learning algorithms. Lysines with a predicted score greater than 0.62 were considered likely to be ubiquitinated. The corresponding GOT1 FASTA sequence, prediction results, and scoring criteria are provided in Supplementary Table 4.

### Cell lines and transfection

The human intrahepatic cholangiocarcinoma cell lines (HUCCT1, HCCC9810, RBE, TFK-1, CCLP-1, ETK-1) and immortalized human intrahepatic cholangiocarcinoma epithelial cells (HIBEC) were obtained from Wuhan PROCELL Biotechnology Co., Ltd. (Wuhan, China). Each cell line is within 10 generations and has undergone mycoplasma testing and cell identification without any contamination. HUCCT1 and HCCC9810 cells, as well as RBE cells in logarithmic growth phase, were seeded and adhered approximately 1 × 10^5^ in a six well plate. After 24 h, the cells adhered well with a cell confluence of about 20–40%. Using the constructed ANXA1/USP5/GOT1 knockdown or overexpression virus solution (Jikai Gene, Shanghai), lentivirus information can be found in Supplementary Table 3, Serum containing culture and A/P solution were mixed in a certain proportion and added to the original culture medium. After 16 h, the culture medium was changed. When the cells grew to about 70–80%, a certain concentration of puromycin was added to the 6-well plate, and screening was continued for 3 days. After no cell death was observed, the puromycin concentration was reduced to its original level of concentration of 1/2 or 1/3 and maintained it. Then reverse transcription quantitative PCR (RT-qPCR) and Western blotting were used to verify the infection status for subsequent experiments.

### RT-qPCR

Total RNA was extracted from ICC tissues and ICC cells using a total RNA isolation kit (Yishan, Shanghai), and cDNA was synthesized using PrimeScript RT reagent (TAKARA, JAPAN). qPCR was performed using the SYBR Green PCR kit (TAKARA) using the RealTime PCR system (Thermo Fisher, Massachusetts, USA), and mRNA expression levels were analyzed using the 2^−ΔΔCt^ method. Use tubulin as an internal control. The primers used for this analysis are listed in Supplementary Table 3.

### Western blot analysis

Proteins were extracted from intrahepatic cholangiocarcinoma tissues and cells using RIRP lysis buffer (Cat: R0010, Solarbio, Beijing). Proteins were separated by 10% SDS-PAGE (Yaase, Shanghai, China) and transferred to PVDF membrane (Milipore, Massachusetts, USA). Seal the membrane in blocking buffer (Yaase) for 30 min, incubate overnight with primary antibody in dilution buffer at 4 C, and then incubate with secondary antibody. Immunoblot imaging was performed using ECL kit (Meilun, Shanghai, China) and chemiluminescence gel imaging system (BIO-RAD, USA). The antibodies used in this assay are listed in Supplementary Table 3 of the attached document.

### Cell proliferation assay

For the Cell Counting Kit-8 (CCK-8) assay, the transfected cells were cultured in a 96 well plate (500 cells/well). At designated time points (0, 1, 2, 3 days), add 10 µ L of CCK-8 solution to each well and incubate the plate at 37 ℃ for 2 h. Measure the absorbance (450 nm) of each well using Multiskan GO (Thermo Fisher). For the determination of 5-ethynyl-20-deoxyuridine (EdU), transfected cells were cultured in a 96 well plate (20000 cells/well) for 2 days, and 50 mM EdU (Yarase, China) was added to each well. The plate was incubated at 37 C for 0.5 h. Then fix the cells, permeabilize them, and treat them with 200 µ L DAPI to stain the nuclei. Count the number of cells in five randomly selected areas of each well to determine the proportion of EdU positive cells. For plate cloning, the transfected cells were seeded in a 6-well plate (500 cells/well) and incubated for 14 days in 2 mL of culture medium. The proliferating colonies were fixed with alcohol and stained with crystal violet.

### Co-IP and mass spectrometry (MS)

To investigate protein interactions, cell lysates were prepared and incubated with magnetic beads conjugated to specific antibodies for Co-IP. Following incubation, the magnetic beads were washed to eliminate unbound proteins, and the bound protein complexes were eluted. Subsequently, the eluted proteins were separated by SDS-PAGE, and bands of interest were excised for trypsin digestion. The resulting peptides were analyzed by mass spectrometry, and interacting proteins were identified through database searches, thereby providing a deeper understanding of molecular associations within the cell lysates. The raw MS data are provided in Supplementary Table 2.

### Ubiquitination IP assay

Antibodies to FLAG (labeled as GOT1) were employed to immunoprecipitate the corresponding experimental treatment groups in order to isolate specific protein complexes. Protein G magnetic beads were utilized to capture the antibody-protein complexes from cell lysates. Following washing, the precipitated proteins were analyzed by Western blotting to verify the level of ubiquitination.

### IHC staining and scoring

Tumor sections fixed with formalin and embedded in paraffin (FFPE) were subjected to immunohistochemical (IHC) staining to assess protein expression in vivo. The slides were dewaxed, rehydrated, and subjected to antigen retrieval. Primary antibodies targeting ANXA1 and GOT1 were applied, followed by incubation with a secondary antibody conjugated to horseradish peroxidase (HRP). Staining was performed using a chromogenic substrate, and the slides were counterstained with hematoxylin. The intensity and distribution of staining were scored by two independent pathologists in a blinded manner (IRS = staining intensity × proportion of positive cells, with a scoring range of 0–12), IRS is a semiquantitative method combining staining intensity and the percentage of positively stained cells. Specifically, staining intensity was scored as follows: 0 (no staining), 1 (weak), 2 (moderate), and 3 (strong). The percentage of positive cells was scored as: 0 (0%), 1 (≤ 10%), 2 (11–50%), 3 (51–80%), and 4 (≥ 81%). The final IRS was calculated as: IRS = intensity score × proportion score. Based on the IRS values, we defined: ANXA1-Low: IRS ≤ 3;ANXA1-High: IRS ≥ 4 [21, 27].

### Immunofluorescence (IF)

#### Cellular immunofluorescence

For immunofluorescence staining, cells were fixed with 4% paraformaldehyde, permeabilized with 0.1% Triton X-100, and blocked to reduce non-specific antibody binding. Primary antibodies targeting ANXA1、USP5 and GOT1 were applied, followed by incubation with fluorescently labeled secondary antibodies. The cell nuclei were counterstained with DAPI to observe cell morphology. Fluorescence signals were captured using a confocal microscope, and the images were processed with appropriate software for colocalization analysis.

#### Multiplex immunofluorescence (mIF)

ICC Tissue sections were deparaffinized with xylene, rehydrated through graded ethanol solutions, fixed in 10% neutral formalin, and permeabilized. Antigen retrieval was performed by boiling sections in antigen retrieval solution using microwave heating, followed by cooling at room temperature. Sections were then treated with 3% hydrogen peroxide and blocked. Samples were sequentially incubated with primary antibodies and HRP-conjugated secondary antibodies, followed by incubation with fluorescent dyes for signal amplification. Each staining round included microwave processing, blocking, and antibody incubation steps. After completing multiple staining rounds, nuclei were stained with DAPI, and sections were mounted with anti-fluorescence quenching mounting medium. Images were acquired and analyzed using fluorescence microscopy. All procedures were performed according to the manufacturer’s instructions of the multiplex immunofluorescence staining kit (abs50012, Absin, Shanghai, China).

### Nude mouse tumorigenicity assay

Four-week-old BALB/c nude mice (female) were purchased from Tianqin Biotechnology Co., Ltd. (Changsha, Hunan Province). The mice were housed in a specific pathogen-free (SPF) environment and randomly divided into groups based on body weight, fed with standard feed or Glutamine-deficient diet (Trofi, Guangdong, China). All animal experiments were conducted in accordance with the animal protection and use regulations of Guizhou Medical University and approved by the Animal Ethics Committee. Transfected HUCCT1/HCCC9810/RBE cells (1 × 10^6^ cells/100 ml) were subcutaneously injected into the flank of six-week-old BALB/c nude mice according to the experimental design (sh-ctrl, sh-ANXA1#1, vector, ANXA1-OE, sh-ANXA1#1 + oe-GOT1, with 5 mice randomly assigned to each group). As for the glutamine deprivation diet, after tumor formation, we gave equal amounts of normal feed and glutamine-deficient feed according to the grouping. All mice were examined every four days (with measurements of tumor volume and body weight), and euthanasia was performed after 28 days, with subcutaneous tumors removed.

### Glutamine uptake assay

Following the manufacturer’s instructions, a colorimetric assay kit (Glutamine Assay Kit, ab197011, Abcam, Cambridge, UK) was utilized to determine the glutamine uptake levels of HUCCT1, HCCC9810, RBE cells and under corresponding treatment conditions. The uptake ratio was defined as C = A/B%. For targeted inhibitors, the respective glutamine levels were measured 24 h after the addition of the corresponding inhibitor. All measurements were conducted after cell attachment. (OD 450 nm)

### Glutamate production assay

According to the manufacturer’s instructions, a colorimetric assay kit (Glutamate Assay Kit, ab83389, Abcam, Cambridge, UK) was used to detect the relative levels of glutamate in HUCCT1, HCCC9810, and RBE cells with stable ANXA1 overexpression or knockdown. Detection was performed 24 h after the addition of targeted inhibitors. (Detection was OD 450 nm)

### Aspartate production assay

Following the manufacturer’s instructions, a colorimetric test kit (Aspartate Assay Kit, E-BC-K849-M, Elabscience, Wuhan, China) was used to detect the relative levels of aspartate in HUCCT1, HCCC9810, and RBE cells with stable ANXA1 overexpression or knockdown. Detection was performed 24 h after the addition of targeted inhibitors. (Detection was OD 450 nm)

### Detection of reactive oxygen levels

According to the manufacturer’s instructions, 2,7-dichlorofuorescindiacetate (DCFH-DA) (Elabscience, E-BC-K138-F, China) was used to detect the ROS levels of nude mouse subcutaneous tumor tissues of equal weight and in vitro group treatment (HUCCT1, RBE cell lines). The accumulation of DCF was measured at an excitation wavelength of 510 nm and an emission wavelength of 530 nm using a fluorescence microplate reader, and statistical analysis was performed.

### Detection of GSH levels

According to the manufacturer’s instructions, dithiodinitrobenzoic acid (DTNB) (Elabscience, E-BC-K030-M, China) was used to detect the GSH levels of nude mouse subcutaneous tumor tissues of equal weight and in vitro group treatment (HUCCT1, RBE cell lines). The microplate reader was used at a wavelength of 405 nm to use colorimetric quantitative determination of GSH content, and statistical analysis was performed.

### Proximity ligation assay (PLA)

Detection of protein–protein interactions by proximity ligation assay (PLA): Protein–protein interactions among ANXA1, USP5, and GOT1 were detected using the NaveniFlex^®^ Cell MR proximity ligation assay (Navinci Diagnostics, Sweden) in human intrahepatic cholangiocarcinoma cell lines HUCCT1 and HCCC9810. All primary antibodies were rabbit or mouse polyclonal antibodies obtained from Proteintech and diluted according to the manufacturer’s recommendations for immunocytochemistry. Cells were cultured on sterile glass slides, fixed with 4% paraformaldehyde for 15 min, and permeabilized with 0.1% Triton X-100 for 10 min at room temperature. After blocking with the kit-provided blocking buffer for 1 h at 37 °C in a humidified chamber, cells were incubated with a mixture of two primary antibodies specific for each interaction pair (ANXA1-USP5&ANXA1-GOT1&USP5-GOT1) for 1 h at 37 °C or overnight at 4 °C. After washing with TBS-T, cells were incubated with the NaveniFlex M1 and R2 proximity probes (diluted 1:40) for 1 h at 37 °C, followed by warm TBS-T washes as recommended. Proximity ligation and rolling circle amplification were performed using the two-step reaction system according to the manufacturer’s protocol. Briefly, Reaction 1 (ligation) was prepared by diluting Buffer 1 (1:5) and Enzyme 1 (1:40) in water and incubated at 37 °C for 30 min. Reaction 2 (amplification) was prepared similarly using Buffer 2 Red (Cy3 channel) and Enzyme 2, and incubated at 37 °C for 90 min in the dark. Nuclei were counterstained with DAPI. Following sequential washes in 1× TBS and 0.1× TBS, slides were mounted using antifade mounting medium. Fluorescence imaging was performed using a Nikon AX confocal microscope (Nikon, Japan) with NIS-Elements 5.4 software. Images were acquired under a 40× dry objective lens. The Cy3 signal (representing protein proximity events) and DAPI-stained nuclei were analyzed using ImageJ (NIH, USA).

### Quantification and statistical analysis

Data analysis was performed using SPSS Statistics (version 19.0; SPSS, New York, USA) and GraphPad Prism 9.5 (GraphPad Software, California, USA). MATLAB Compiler Runtime (MCR, version: R2009a MCR 7.10) is used in ubiquitination site prediction. Two-tailed Student’s t-tests 、One-way ANOVA、two-way ANOVA were used to assess differences between two groups and more. Chi-square tests were used to determine the correlation between ANXA1 expression levels and clinical pathological parameters of ICC patients. Spearman’s correlation analysis was performed to assess the relationship between ANXA1 and GOT1 expression levels. Kaplan-Meier analysis was used to analyze OS and DFS. All assays were conducted at least three times, and data are presented as the mean ± SD. *P* < 0.05 was considered statistically significant (*, *P* < 0.05; **, *P* < 0.01; ***, *P* < 0.001).

## Results

### Identification of genes highly associated with metabolism and prognosis of ICC using bioinformatics

**Fig. 2:**
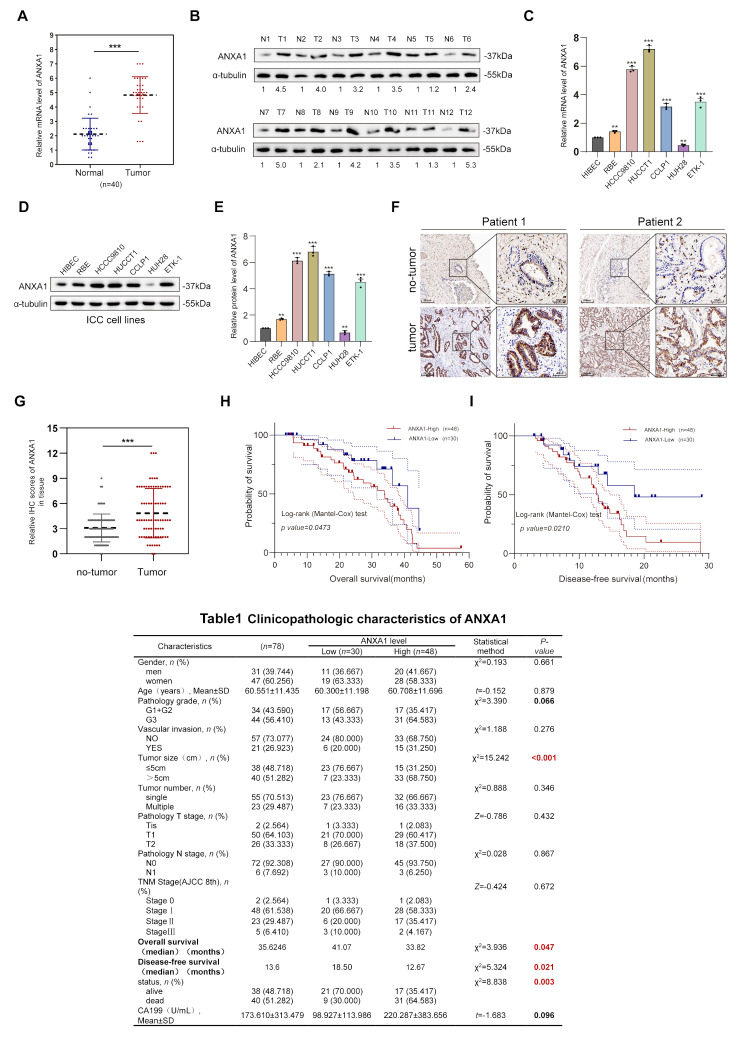
ANXA1 is highly expressed in ICC tissues and cells and is associated with poor prognosis. **A**: mRNA expression levels of ANXA1 in 40 pairs of ICC tumor and normal tissues(*n* = 40). **B**: Protein levels of ANXA1 in 12 pairs of ICC tumor and normal tissues. **C**-**E**: mRNA and protein expression of ANXA1 in ICC cell lines and normal biliary epithelial cell lines(*n* = 3). **F**-**G**: Representative immunohistochemical images of ANXA1 in 78 pairs of ICC tissues and corresponding normal tissues (scale bar 250 μm) and statistical graph of immunohistochemical scoring (*n* = 78, IHC score 0–12). **H**-**I**: Kaplan-Meier analysis of overall and disease-free survival in low and high ANXA1 expression groups. Data are representative of three **(A**,** C**,** E**) independent experiments. Unpaired two-tailed Student’s t-tests (**A**,** G**); One-way ANOVA with Dunnett’s test (**C**,** E**); Log-rank (Mantel–Cox) test (**H**,** I**). **, *p* < 0.01; ***, *p* < 0.001. Data are mean ± SD

To investigate the metabolic landscape of intrahepatic cholangiocarcinoma, we analyzed RNA-seq data from publicly available ICC datasets, including TCGA-CHOL (*n* = 32), NODE (OEP001105, *n* = 244), GSE26566 (*n* = 104), GSE107943 (*n* = 30), E-MTAB-6389 (*n* = 78), GSE107100 (*n* = 24), GSE89747 (*n* = 83), and GSE32225 (*n* = 149) (Fig. 1A). Using normalized transcriptome sequencing data and 76 KEGG metabolic pathways (*KEGG: Kyoto Encyclopedia of Genes and Genomes*), we conducted GSVA and performed consensus clustering analysis (consensus matrix, k = 2). This analysis classified the samples into two major metabolic subtypes: Cluster1 and Cluster2 (Fig. 1B), with distinct clinicopathological characteristics (Fig. 1C). Patients in Cluster2 demonstrated significantly poorer clinical outcomes compared to those in Cluster1 (Fig. 1D). To pinpoint specific metabolic differences, we performed pathway-based metabolic type differential analysis. Cluster2, associated with poor prognosis, exhibited upregulation of amino acid metabolism, fatty acid metabolism, and glycolysis. In contrast, Cluster1 showed upregulation of nucleotide metabolism and MAPK signaling pathways (Fig. 1E). Therefore, we further conducted differential gene analysis (Fig. 1F), and based on the differential genes, performed GO and KEGG enrichment analyses, finding that it was mainly related to intracellular amino acid metabolism, fatty acid transport, glutamate, aspartate, and glutamine metabolism pathways, and glycolysis (Supplementary Fig. S1A). These findings underscore the critical role of amino acid and lipid metabolism in influencing ICC prognosis. Subsequently, we identified 23 candidate genes as potential metabolism-related markers by intersecting differentially expressed genes (DEGS) from Clusters1 and Clusters2, DEGs from the TCGA dataset, and DEGs from the GSE26566 dataset (Fig. 1G). A single-gene Cox regression analysis using TCGA-CHOL Dataset identified ANXA1 as a target gene with a high metabolic risk score and significant prognostic relevance in ICC (Fig. 1H). ANXA1 may represent a potential therapeutic target. Further validation across multiple datasets, including TCGA-CHOL, demonstrated elevated ANXA1 expression in tumor tissues compared to controls, with statistically significant differential expression (Fig. 1I, Supplementary Fig. S1B-C). Moreover, a prognostic analysis revealed significant survival differences between high- and low-expression groups in the NODE Dataset (OEP001105, *n* = 255) (Fig. 1J). Single gene GSEA analysis in the TCGA-CHOL and NODE datasets with typical ICC features showed that high expression of ANXA1 was significantly both enriched in metabolic pathways such as alanine, aspartate, and glutamate metabolism, glycolysis, and gluconeogenesis (Fig. 2K).

### ANXA1 is highly expressed in ICC tissues and cells and is associated with poor prognosis

To evaluate the expression of ANXA1 in ICC, we analyzed frozen tissue samples collected from 40 patients with ICC. RT-qPCR analysis revealed that ANXA1 expression in tumor tissues was significantly higher compared to normal tissues (Fig. 2A). Western blot analysis further confirmed elevated ANXA1 expression in ICC tissues relative to adjacent normal tissues (Fig. 2B). Next, we analyzed ANXA1 expression in 31 ICC cell lines using data from public databases(https://depmap.org/portal/) (Supplementary Fig. S2A). Most ICC cell lines exhibited high levels of ANXA1 expression, which was corroborated by consistent mRNA and protein expression levels in these cell lines (Fig. 2C-E). We further investigated ANXA1 expression in 78 paired ICC tumor and adjacent normal tissue samples. The results consistently demonstrated significantly higher ANXA1 expression in tumor tissues than in normal tissues (Fig. 2F). This finding was validated through immunohistochemical scoring, which confirmed the overexpression of ANXA1 in ICC tissues (Fig. 2G). Notably, survival analysis revealed that patients with high ANXA1 immunohistochemical (IHC) scores (high expression group) had significantly lower overall survival (OS) and disease-free survival (DFS) rates compared to patients in the low-expression group (Fig. 2H-I). Clinicopathological data from the 78 patients (Fig. 2. Table 1) indicated that ANXA1 expression was significantly associated with tumor size and survival status even if no statistically significant difference was observed in TNM staging, suggested that the high expression ANXA1 is a potential marker for poor prognosis in ICC patients.

### ANXA1 promotes ICC cell proliferation and growth in vitro and vivo

**Fig. 3:**
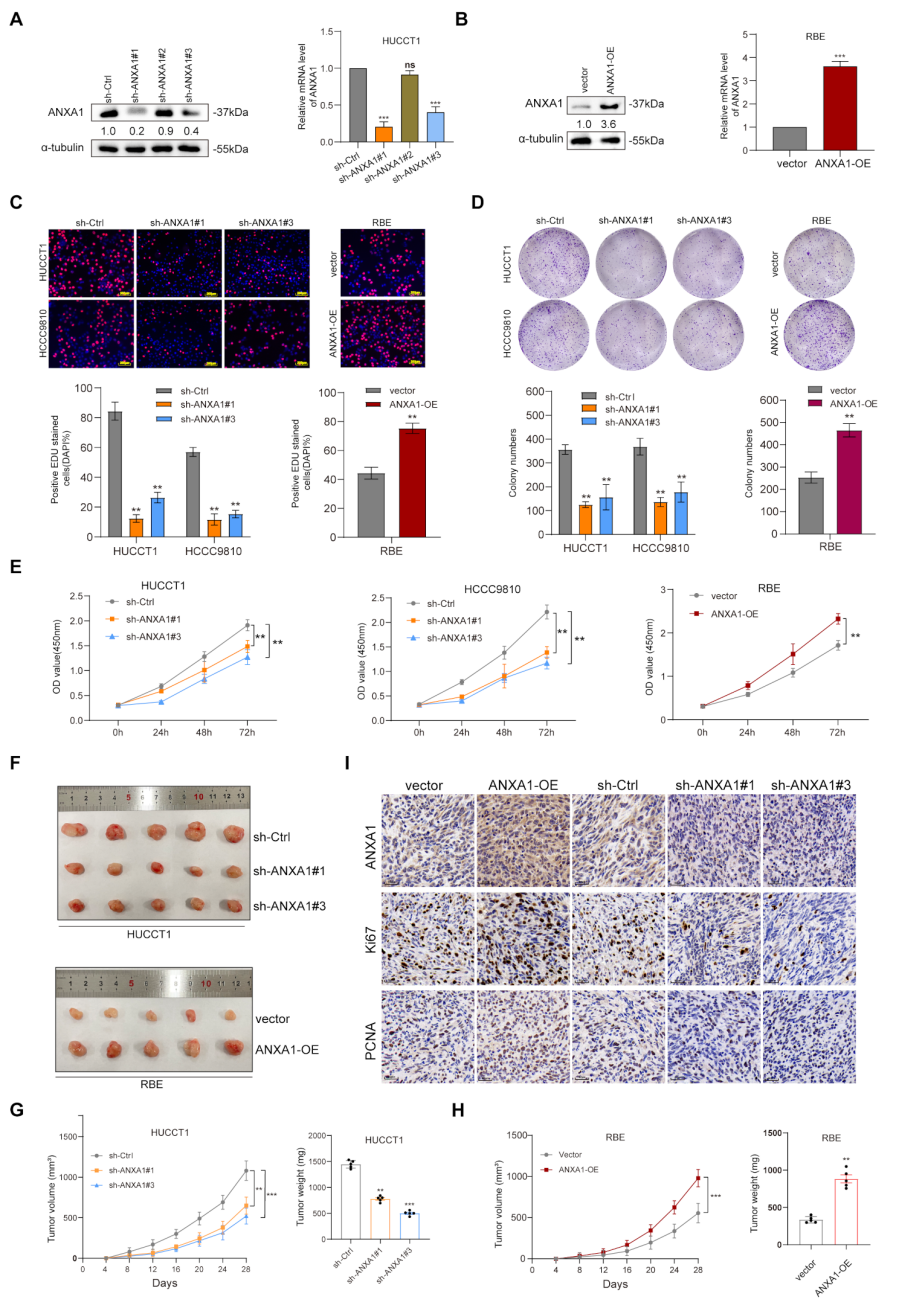
ANXA1 promotes ICC cell proliferation and growth in vitro and vivo. **A**-**B**: Validation of ANXA1 knockdown in HUCCT1 cell lines and overexpression in RBE cell line by western blot and RT-PCR. **C**: EDU assay to detect the proliferative capacity of ANXA1 knockdown or overexpression. **D**: Plate cloning assay to detect the proliferative capacity of ANXA1 knockdown HUCCT1 and HCCC9810 cells and RBE overexpression. **E**: CCK-8 assay to detect the proliferative capacity of ANXA1 knockdown HUCCT1 and HCCC9810 cells and RBE overexpression. **F**: Xenograft mouse model of tumors from HUCCT1 cells with ANXA1 knockdown and RBE cells with ANXA1 overexpression. **G**: Volume and weight changes of xenograft tumors from HUCCT1 cells with ANXA1 knockdown. **H**: Volume and weight changes of xenograft tumors from RBE cells with ANXA1 overexpression. **I**: Immunohistochemical staining of KI67 and PCNA in mouse subcutaneous xenograft tumor tissues. Data are representative of three (**A**,** B**,** C**,** D**,** E**) or five (**G**,** H**) independent experiments. Unpaired two-tailed Student’s t-tests (**B**,** C(right)**,** D(right)**,** H(right)**); One-way ANOVA with Dunnett’s test (**A**,** C(left)**,** D(left)**,** G(right)**); two-way ANOVA (**E**,** G(left)**,** H(left)**); Log-rank (Mantel–Cox) test (**H**,** I**). **, *p* < 0.01; ***, *p* < 0.001; ns, not significant. Data are mean ± SD

To establish appropriate experimental models, we selected two cell lines, HUCCT1 and HCCC9810, for ANXA1 knockdown based on their high expression levels observed in our previous analysis (Fig. 2C-E). In addition, the RBE cell line was used to construct an ANXA1-overexpressing model. We successfully generated ANXA1-knockdown HUCCT1 and HCCC9810 cell lines, as well as ANXA1-overexpressing RBE cells, and confirmed the modifications at the RNA level (Fig. 3A-B). The EDU assay demonstrated that ANXA1 knockdown significantly reduced cell proliferation, whereas overexpression of ANXA1 markedly enhanced proliferation compared to the empty vector group (Fig. 3C). These findings were further validated using a cell counting kit-8 (CCK-8) assay, which confirmed that the proliferation capabilities of ICC cells were significantly influenced by ANXA1 expression (Fig. 3D-E). To further investigate the role of ANXA1 in tumor growth in vivo, we constructed a subcutaneous xenograft tumor model. Tumor growth was significantly inhibited in mice injected with ANXA1-knockdown HUCCT1 and HCCC9810 cells compared to the control group (**p < 0.01). Conversely, tumor growth was significantly accelerated in mice injected with ANXA1-overexpressing RBE cells (***p < 0.001) (Fig. 3.F-H). Immunohistochemical analysis of the xenografts showed that proliferation-related markers, such as Ki67 and PCNA, were reduced in the knockdown group and increased in the overexpression group (Fig. 3I). These findings indicate that ANXA1 positively regulates ICC progression both in vitro and in vivo.

### ANXA1 interacts with GOT1 and regulates its protein expression

**Fig. 4:**
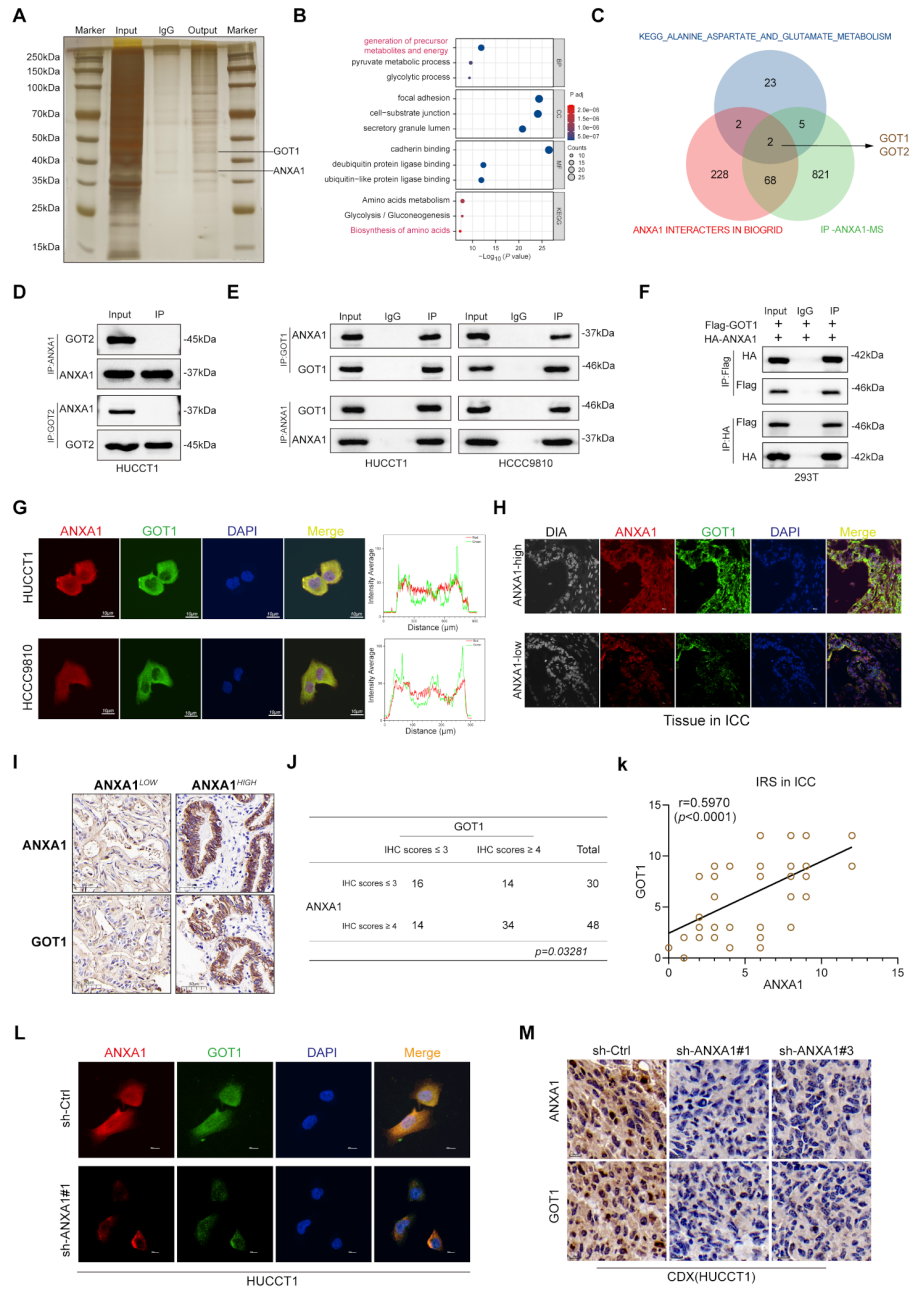
ANXA1 interacts with GOT1 and regulates its protein expression. **A**: Silver staining image before mass spectrometry analysis (IP-ANXA1). **B**: Enrichment analysis of potential proteins associated with ANXA1 (KEGG and GO). **C**: Venn diagram based on public database MsigDB, Amino acid metabolism, BIOGRID, and mass spectrometry analysis intersection. **D**: CO-IP detection of the interaction between ANXA1 and GOT2 in HUCCT1 cells. **E**: CO-IP detecting the interaction between ANXA1 and GOT1 in HUCCT1 and HCCC9810 cells endogenously. **F**: CO-IP detecting the interaction between ANXA1 and GOT1 in 293T cells exogenously. **G**: Immunofluorescence detecting the co-localization of ANXA1 and GOT1 in HUCCT1 and HCCC9810 cells and fluorescence intensity statistics. **H**: Immunofluorescence detecting the co-localization of ANXA1 and GOT1 in typical ICC tissues. **I**: Representative immunohistochemical (IHC) staining of ANXA1 and GOT1 in tumor tissues. **J**: A contingency table was constructed based on IHC scores to assess the expression distribution of ANXA1 and GOT1 in 48 cholangiocarcinoma tissue samples(chi-square test). **K**: Correlation analysis of ANXA1 and GOT1 expression in intrahepatic cholangiocarcinoma (ICC) tissues based on immunoreactive scores (IRS)(Spearman’s correlation). **L**: Immunofluorescence detecting the expression of GOT1 in HUCCT1 cells with ANXA1 knockdown. **M**: Immunohistochemical staining to detect the protein expression level of GOT1 in subcutaneous tumor tissues of HUCCT1 cells with ANXA1 knockdown

To explore the specific mechanisms by which ANXA1 contributes to the development of ICC, The previous bioinformatics analysis showed that high expression levels of ANXA1 are significantly correlated with amino acid metabolism and glucose metabolism in tumor. we conducted immunoprecipitation (Co-IP) followed by mass spectrometry (IP-MS) (Fig. 4A), identifying 900 proteins (Supplementary Table 2). The identified proteins were significantly enriched in pathways related to generation of precursor metabolites and energy, biosynthesis of amino acids, and amino acid metabolism (Fig. 4B). To pinpoint potential metabolic targets, we integrated data from public databases (https://thebiogrid.org/) with KEGG metabolic pathways for alanine, aspartate, and glutamate metabolism related genes. This analysis identified GOT1 and GOT2 as intersecting candidates (Fig. 4C), providing theoretical support for ANXA1’s involvement in amino acid metabolism in ICC. GOT1 and GOT2 are cytosolic and mitochondrial isoforms of glutamate aminotransferase, respectively. GOT2 facilitates the conversion of glutamine-derived glutamate into aspartate within mitochondria, with portions entering the citric acid cycle or exiting mitochondria. GOT1 subsequently converts oxaloacetate and α-ketoglutarate (α-KG) into intermediates that fuel tumor cell proliferation.

To verify whether ANXA1 directly regulates GOT1 or GOT2, immunoprecipitation experiments confirmed that GOT2 does not interact with ANXA1 (Fig. 4D). However, ANXA1 unexpectedly interacted with GOT1 endogenously (HUCCT1, HCCC9810) and exogenously (HEK293T) (Fig. 4E-F). Immunofluorescence staining further demonstrated that ANXA1 and GOT1 colocalize in the cytoplasm (Fig. 4G), further PLA experiments demonstrated that ANXA1 directly interacts with GOT1 in the cytoplasm (Supplementary Fig. S3A). This colocalization was also observed in ICC tissue samples, confirming that high ANXA1 expression correlates with increased binding to GOT1 (Fig. 4H). Immunohistochemical analysis indicated a positive correlation between ANXA1 and GOT1 expression levels (Fig. 4I). Immunoreactive Score (IRS) statistical analysis showed that the IRS of ANXA1 and GOT1 were positively correlated, which was statistically significant in ICC tissue (Fig. 4J-K). This was consistently supported by immunofluorescence staining in ANXA1-knockdown ICC cell lines, which confirmed reduced GOT1 expression (Fig. 4L, Supplementary Fig. S3B). Similarly, we performed immunohistochemical staining on subcutaneous tumor tissue constructed using the HUCCT1 knockdown cell line and observed the same changes (Fig. 4M).

### ANXA1 regulates glutamine metabolism via deubiquitination and protein stability of GOT1

**Fig. 5:**
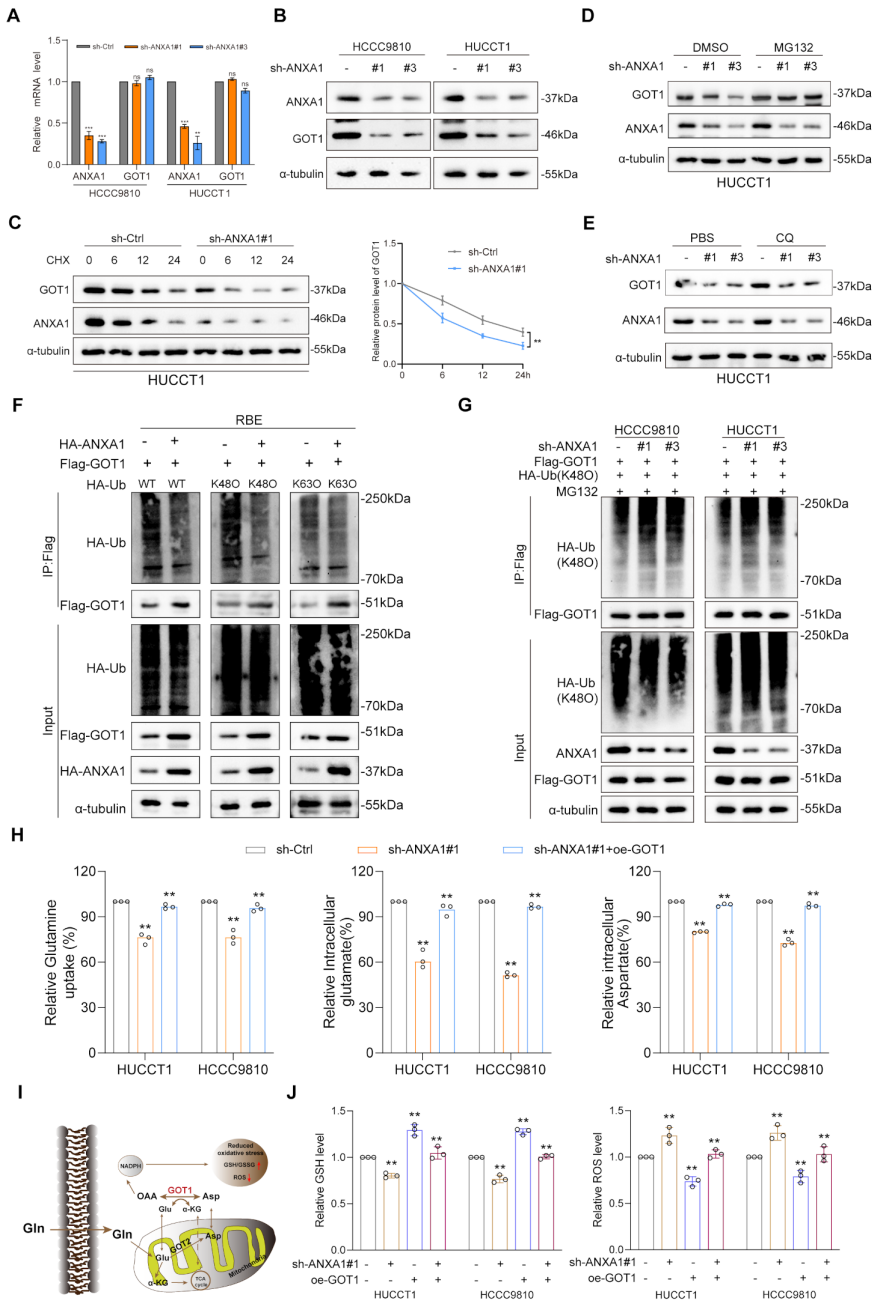
ANXA1 regulates glutamine metabolism via deubiquitination and protein stability of GOT1. **A**: RT-PCR detecting the mRNA levels of GOT1 after knockdown of ANXA1 in HUCCT1 and HCCC9810 cells. **B**: Western blot detecting the expression of GOT1 in HUCCT1 and HCCC9810 cells with ANXA1 knockdown. **C**: IB of GOT1, ANXA1, and α-tubulin in HUCCT1 cells transduced with sh-ANXA1#1 or sh-control after CHX treatment (100 µg/ml) for the indicated times (left). A graph showing normalized GOT1 levels is also shown (right). **D**-**E**: IB of GOT1, ANXA1, and α-tubulin in HUCCT1 cells transduced with sh-control or sh-ANXA1 after treatment with MG132 (10 μm, top) or CQ (50 μm, bottom). **F**: IP (using anti-Flag antibody) and IB of HA-Ub, Flag, ANXA1, α-Tubulin, and GOT1 in RBE cells transfected with the indicated plasmids.(k48O refers to ubiquitin expression vector mutant plasmid that retains only the K48 site, k63O refers to ubiquitin expression vector mutant plasmid that retains only the K63 site). **G**: IP (using anti-Flag antibody) and IB of HA-Ub(k48O), Flag, ANXA1, α-Tubulin, and GOT1 in HUCCT1 and HCCC9810 cells transfected with the indicated plasmids after treatment with MG132 (10 μm, 6 h). **H**: Glutamine uptake and glutamate/ aspartate levels were examined in HUCCT1 and HCCC9810 cells with stable of knockdown ANXA1 or overexpress GOT1. **I**: Mechanism diagram of GOT1-mediated glutamine metabolism. **J**: GSH and ROS levels were examined in HUCCT1 and HCCC9810 cells with stable of knockdown ANXA1 or overexpress GOT1. Data are representative of three (**A**,** C(right)**,** H**,** J**) independent experiments. One-way ANOVA (**A**,** H**,** J**); Two-way ANOVA (**C(right)**). **, *p* < 0.01; ***, *p* < 0.001; ns, not significant. Data are mean ± SD

Given the regulatory relationship between GOT1 and ANXA1, we explored the specific mechanisms underlying this interaction. We next explored whether ANXA1 regulates GOT1 at the transcriptional or protein level. Analysis of public TCGA-CHOL Dataset revealed no regulatory relationship between ANXA1 and GOT1 at the transcriptional level, a finding that was validated in ICC cell lines (Fig. 5A and Supplementary Fig. S4A-B). However, protein level analysis indicated that GOT1 expression is modulated by ANXA1. In cell lines with ANXA1 knockdown, GOT1 protein levels were significantly reduced, whereas ANXA1 overexpression increased GOT1 protein levels (Fig. 5B and Supplementary Fig. S4C).

To elucidate how ANXA1 influences GOT1 protein expression level, we observed that GOT1 protein levels decreased upon ANXA1 knockdown. Cycloheximide (CHX) treatment further demonstrated that ANXA1 knockdown accelerated the degradation of endogenous GOT1 (Fig. 5C and Supplementary Fig. S4D). These results suggest that ANXA1 stabilizes GOT1 protein by inhibiting its degradation in intrahepatic cholangiocarcinoma cells. We next investigated whether ANXA1 modulates GOT1 degradation through the ubiquitin-proteasome or autophagy-lysosome pathway. Treating cells with the proteasome inhibitor MG132 reversed the decrease in GOT1 protein levels induced by ANXA1 knockdown, whereas the lysosome inhibitor (CQ) did not (Fig. 5D-E). These results indicate that ANXA1 affects GOT1 stability through the ubiquitin-proteasome pathway. To confirm this, we examined GOT1 ubiquitination in ANXA1-knockdown cells. Knockdown of ANXA1 increased K-48 chain-mediated, but not K-63 chain-mediated, ubiquitination of GOT1 (Fig. 5F-G). These findings suggest that ANXA1 regulates the stability of GOT1 by regulating its ubiquitination.

Next, we further explored whether ANXA1 could affect GOT1-mediated glutamine metabolism and its mechanism of promoting tumor cell proliferation. To explore this, we measured aspartate and glutamate production in cell lysates from ANXA1-knockdown cholangiocarcinoma cells, as well as tissue lysates from subcutaneous xenograft tumors in mice. We also assessed glutamine uptake and consumption in intrahepatic cholangiocarcinoma cell lines. In ANXA1-knockdown cells, the levels of aspartat and glutamate were significantly reduced compared to controls, overexpression of GOT1 in ANXA1 knockdown cells reversed the stabilizing effect of ANXA1 knockdown on GOT1, a trend also observed in the xenograft tumor model (Fig. 5H and Supplementary Fig. S4E). Glutamine uptake was similarly decreased. Conversely, ANXA1 overexpression increased GOT1-related metabolite levels and enhanced glutamine uptake, knockdown of GOT1 can weaken this ability in vivo and in vitro (Supplementary Fig. S4F-G), thereby supplying essential precursors for tumor biosynthesis and maintaining energy homeostasis, further increasing the resilience of tumor cells to oxidative stress (Supplementary Fig. S4H).Therefore, cell experiments have shown that knocking down or overexpressing ANXA1 can reduce GSH or increase ROS levels, while overexpression or knocking down GOT1 can reverse this change. In summary, in ICC cells with high expression of ANXA1, ANXA1 further increases the adaptability of Glutamine metabolism mediated oxidative stress by stabilizing ubiquitin proteasome mediated GOT1, thereby promoting tumor growth and proliferation (Fig. 5I-J). However, the mechanism by which ANXA1 stabilizes GOT1 through the ubiquitin proteasome is still unclear.

### ANXA1 recruits deubiquitinating enzyme USP5 to deubiquitinate and stabilize GOT1

**Fig. 6:**
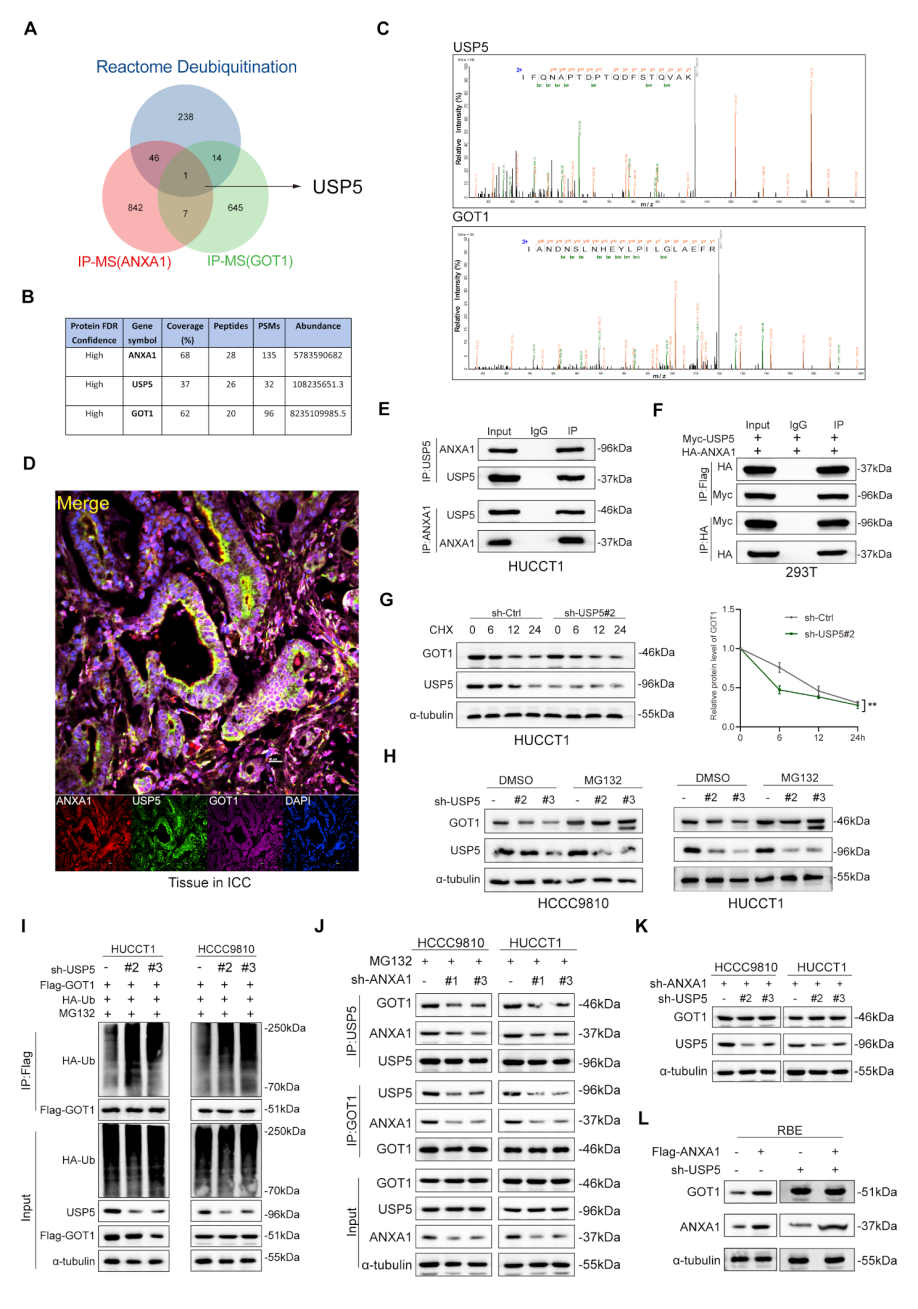
ANXA1 Recruits Deubiquitinating Enzyme USP5 to Deubiquitinate and Stabilize GOT1. **A**-**C**: Venn diagram based on IP-MS (ANXA1 and GOT1) with Molecular Signatures Database(MsigDB) to find deubiquitinating enzymes that stabilize GOT1 and IP-MS analysis as well as secondary mass spectrometry diagrams of USP5 and GOT1. **D**: Multiplex immunofluorescence detecting the co-localization of ANXA1, USP5, and GOT1 in ICC tissues. **E**: CO-IP detecting the interaction between ANXA1 and USP5 in HUCCT1 cells endogenously. **F**: CO-IP detecting the interaction between ANXA1 and USP5 in 293T cells exogenously. **G**: IB of GOT1, ANXA1, and α-tubulin in HUCCT1 cells transduced with sh-USP5 or sh-control after CHX treatment (100 µg/ml)for the indicated times (left). A graph showing normalized GOT1 levels is also shown (right). (Mean values (*n* = 3) ± s.d. two-way ANOVA, **p < 0.01.). **H**: IB of GOT1, USP5, and α-Tubulin in HUCCT1 and HCCC9810 cells transduced with sh-control or sh-USP5 after treatment with MG132 (10 μm, 6 h). **I**: IP with anti-Flag antibody and IB of HA-Ub, Flag-GOT1, USP5, and α-Tubulin in HUCCT1 and HCCC9810 cells transfected with the indicated plasmids after treatment with MG132 (10 μm, 6 h). **J**: IP (using anti-USP5 or anti-GOT1 antibody) and IB of GOT1, ANXA1, and USP5 in HUCCT1 and HCCC9810 cells transduced with sh-control or sh-ANXA1 after treatment with MG132 (10 μm, 6 h). **K**:IB of GOT1, USP5, and α-Tubulin in HUCCT1 and HCCC9810 cells transduced with sh-control or sh-USP5 and with ANXA1 knockdown. **L**: IB of GOT1, ANXA1, and α-Tubulin in RBE cells transfected with vector or Flag-ANXA1 and with USP5 knockdown

Previously, it was demonstrated that ANXA1 regulates GOT1 protein levels by modulating its ubiquitination. ANXA1 is known to function as either a non-ubiquitin-related ligase or a deubiquitinating enzyme. To elucidate the mechanism underlying this regulation, we used GOT1 as the bait protein in MS (IP-MS for GOT1 & ANXA1) (Supplementary Table 2) and combined the results with deubiquitinating enzyme data from public databases (Reactome deubiquitination https://www.gsea-msigdb.org/gsea/msigdb). This analysis identified USP5 as a potential deubiquitinating enzyme regulating GOT1 deubiquitination (Fig. 6A). Mass spectrometry revealed the detection of three proteins (Fig. 6B), along with their secondary mass spectra, in both immunoprecipitants (IP: ANXA1 or GOT1) (Fig. 6C). These findings were further validated in intrahepatic cholangiocarcinoma tissue (Fig. 6D). To determine whether ANXA1 serves as a scaffold for USP5 to stabilize GOT1, co-immunoprecipitation assays demonstrated that ANXA1 binds to USP5 endogenously and exogenously (Fig. 6E-F). Immunofluorescence analysis confirmed the cytoplasmic colocalization of ANXA1 and USP5 (Supplementary Fig. S5A), meantime PLA experiments demonstrated that ANXA1 directly interacts with USP5 in the cytoplasm (Supplementary Fig. S5B). Additionally, data from the public database Gene Expression Profiling Interactive Analysis (GEPIA: http://gepia.cancer-pku.cn/index.html) showed that ANXA1 does not influence the mRNA expression of USP5 (Supplementary Fig. S5C). Next, we examined whether USP5 affects the protein stability of GOT1 as a specific deubiquitinase. Knockdown of USP5 led to a decrease in GOT1 protein levels without affecting its mRNA expression, whereas USP5 overexpression restored GOT1 protein levels (Supplementary Fig. S5D-G). To assess the impact of USP5 on the half-life of GOT1, we used the protein synthesis inhibitor cycloheximide (CHX) in ICC cell lines with USP5 knockdown. GOT1 protein levels were measured at 0 h, 6 h, 12 h and 24 h. The half-life of GOT1 was significantly shortened in the USP5 knockdown group, as confirmed by protein quantification (Fig. 6G and Supplementary Fig. S5H). This result mirrored the shortened half-life observed in cells with ANXA1 knockdown (Fig. 5C).

To evaluate whether the stabilization of GOT1 by USP5 occurs via deubiquitination, we treated USP5 knockdown cells with a proteasome inhibitor. GOT1 protein levels significantly increased compared to the control group, and proteasome inhibition reversed the degradation of GOT1 caused by USP5 knockdown (Fig. 6H). To further confirm the enzymatic specificity of USP5, we introduced a point mutation (C335A) into its catalytic domain, generating an enzymatically inactive mutant (Myc-USP5-C335A). Co-transfection of Myc-USP5-C335A with Flag-GOT1 in HUCCT1 cells revealed that the mutant USP5 was unable to deubiquitinate or interact with GOT1, further supporting that USP5 exerts its deubiquitination function in a catalytic activity–dependent manner (Supplementary Fig. S5I). Further domain analysis of USP5 and GOT1 confirmed that ANXA1 relies on USP5 and the GOT1 (48–129) truncation to exert its function in glutamine metabolism (Supplementary Fig.S5J-L).In HUCCT1 and HCCC9810 cells treated with MG132, the results showed that the ubiquitination level of GOT1 was significantly increased in the USP5 knockdown groups, suggesting that USP5 may maintain GOT1 stability through a deubiquitination pathway (Fig. 6I). To explore the specific deubiquitination sites involved in the interaction with USP5 on GOT1, we used the MATLAB Compiler Runtime (MCR, version: R2009a MCR 7.10) tool to analyze all lysine residues in the GOT1 amino acid sequence (detailed procedures and results are described in the methods section and supplementary Table 4). We selected four candidate sites (K33, K97, K99 and K325) for site-directed mutagenesis (Supplementary Fig. S6A) and constructed the corresponding plasmids: Flag-GOT1-WT, Flag-GOT1-K33R, Flag-GOT1-K97R, Flag-GOT1-K99R, and Flag-GOT1-K325R. These plasmids were individually transfected into HUCCT1 cells under identical treatment conditions, with or without USP5 knockdown. We observed that in cells transfected with the Flag-GOT1-K97R mutant plasmid, the ubiquitination level of GOT1 remained largely unchanged regardless of USP5 knockdown. In contrast, GOT1 ubiquitination levels were significantly affected in the other mutant and wild-type groups. These results suggest that USP5 specifically recognizes and removes K48-linked ubiquitin chains at the lysine 97 (K97) site of GOT1, thereby stabilizing the protein (Supplementary Fig. S6B). To further confirm the specificity of USP5 as the deubiquitinase responsible for stabilizing GOT1, we transfected HUCCT1 cells with the Flag-GOT1-K97R mutant plasmid and simultaneously knocked down USP5. The results showed that GOT1 protein levels remained largely unchanged regardless of USP5 knockdown, and the trends in both groups were nearly identical (Supplementary Fig. S6C-D). This finding further supports that USP5 specifically regulates GOT1 stability through K97-dependent deubiquitination. Finally, we examined whether ANXA1 recruits USP5 to stabilize GOT1 in ICC cells. In cells with ANXA1 knockdown, the interaction between USP5 and GOT1 was significantly weakened, and the stabilizing effect of USP5 on GOT1 was reduced (Fig. 6J). Further knockdown of USP5 in ANXA1 knockdown cells still cannot alter the protein expression level of GOT1. However, only in the presence of USP5 can the regulatory effect of ANXA1 on GOT1 be significantly observed (Fig. 6K-L). To investigate whether USP5 overexpression can rescue GOT1 expression in the knockdown of ANXA1, HUCCT1 and HCCC9810 cells were transduced with sh-ANXA1 and/or Myc-tagged USP5 (Myc-USP5). Western blot analysis showed that silencing ANXA1 significantly reduced GOT1 protein levels in both cell lines. However, reintroduction of USP5 only partially restored GOT1 expression, suggesting that ANXA1 is not only involved in recruiting USP5 but may also be essential for stabilizing the USP5–GOT1 complex (Supplementary Fig. S6E). Moreover, compared with the vector group, PLA experiments indicate that ANXA1 overexpression enhanced the interaction between USP5 and GOT1 (Supplementary Fig. S6F). These results suggest that ANXA1 acts as a scaffold protein, facilitating USP5-mediated stabilization of GOT1 in intrahepatic cholangiocarcinoma.

### ANXA1 inhibiting combine with glutamine deficiency suppress tumor cells growth and proliferation

**Fig. 7:**
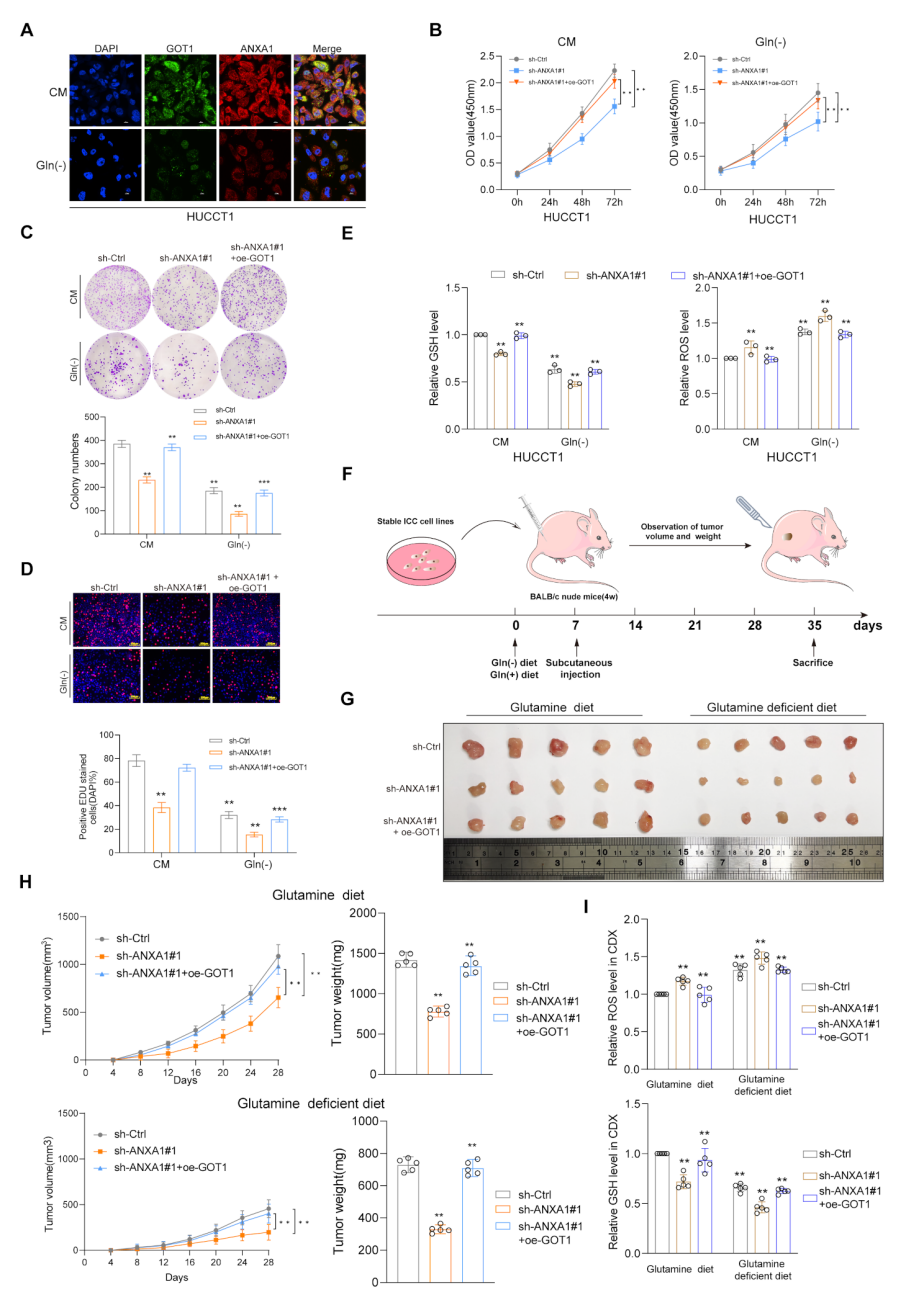
ANXA1 inhibiting combine with glutamine deficiency suppress tumor cells growth and proliferation. **A**: Immunofluorescence co-localization of ANXA1 and GOT1 in HUCCT1 cells cultured in CM or Gln (-). **B**: CCK8 proliferation assay was used to detect the proliferation ability of HUCCT1 cells treated with sh-Ctrl、sh-ANXA1#1、sh-ANXA1#1 + oe-GOT1 in complete medium (CM) and glutamine-deficient Gln (-) medium. **C**: Plate cloning was used to detect the proliferation ability of HUCCT1 cells treated with sh-Ctrl、sh-ANXA1#1、sh-ANXA1#1 + oe-GOT1 in complete medium (CM) and glutamine-deficient (Gln (-)) medium. **D**: EDU assay was used to detect the proliferation ability of HUCCT1 cells treated with sh-Ctrl、sh-ANXA1#1、sh-ANXA1#1 + oe-GOT1 in complete medium (CM) and glutamine-deficient (Gln (-)) medium. **E**:Levels of ROS and GSH was detected in HUCCT1 cells in complete medium (CM) and glutamine-deficient (Gln (-)) medium. **F**: Different groups of stable cell lines were transplanted subcutaneously into nude mice and raised with or without glutamine. **G**: HUCCT1 cells of sh-Ctrl, sh-ANXA1#1, sh-ANXA1#1 + oe-GOT1 were subcutaneously inoculated into nude mice fed with glutamine diet or lacking glutamine diet. **H**: The corresponding tumor volume growth curve and weight are shown. **I**: GSH or ROS levels were examined in CDX with stable of knockdown ANXA1 or overexpress GOT1. Data are representative of three (**B**,** C**,** D**,** H(left)**) or five (**H(right)**,** I**) independent experiments. One-way ANOVA (**C**,** D**,** E**,** H(right)**,** I**); Two-way ANOVA (**B**,** H(left)**). **, *p* < 0.01; ***, *p* < 0.001. Data are mean ± SD

GOT1 is one of the key enzymes involved in glutamine metabolism. The metabolism and products of glutamine support tumor cell growth by maintaining redox balance, which is crucial for the survival and growth of ICC cells. Therefore, we further clarified the role of ANXA1 mediated GOT1 positive regulation in ICC growth. Under complete culture medium conditions, compared with the control group, ANXA1 knockdown cells showed significant growth, and overexpression of GOT1 could almost rescue ANXA1 knockdown tumor inhibition. Similarly, under glutamine deficient culture medium conditions, the growth inhibition effect was more pronounced (Fig. 7A-D). Further intracellular GSH and ROS detection levels showed that ANXA1 knockdown reduced GSH production and increased ROS levels, while overexpression of GOT1 could reverse this change. When glutamine was further depleted, lower GSH levels and higher ROS levels were observed (Fig. 7E). The above indicates that the proliferation of tumor cells is largely dependent on glutamine addiction. In addition, to further explore the tumorigenic ability in vivo, we subcutaneously inoculated ANXA1 knockdown HUCCT1 cells with or without overexpression of GOT1 into nude mice fed a glutamine diet or a glutamine deficient diet. First, the research results were consistent with the proliferation of cells in vitro. Compared with the control group, only ANXA1 knockdown inhibited tumor growth. In the group of ANXA1 knockdown and overexpression of GOT1, tumor formation and growth were restored. Interestingly, in the glutamine deficient diet group, tumor growth was slow and significantly inhibited at the same time (Fig. 7F-G). Further tumor volume growth curves and weight quantification also confirmed this (Fig. 7H). Then we measured glutamine levels within the subcutaneous xenograft tumors in mice with different diet and the results demonstrated that tumors from mice on a glutamine-deficient diet exhibited significantly reduced glutamine concentrations compared to those in the normal diet group (supplementary Fig. S7A). Subcutaneous tumorigenic tissues of mice under different diets showed that inhibition of ANXA1 expression resulted in increased ROS levels and reduced GSH inhibition. However, overexpression of GOT1 could reverse this effect and promote tumor growth in vivo (Fig. 7I). In summary, these results indicate that ANXA1 can regulate the expression of GOT1, promote the adaptive ability of glutamine metabolism mediated tumor associated oxidative stress, further promote tumor growth, and inhibit ANXA1 combined with glutamine deficiency to inhibit tumor growth.

## Discussion

**Fig. 8:**
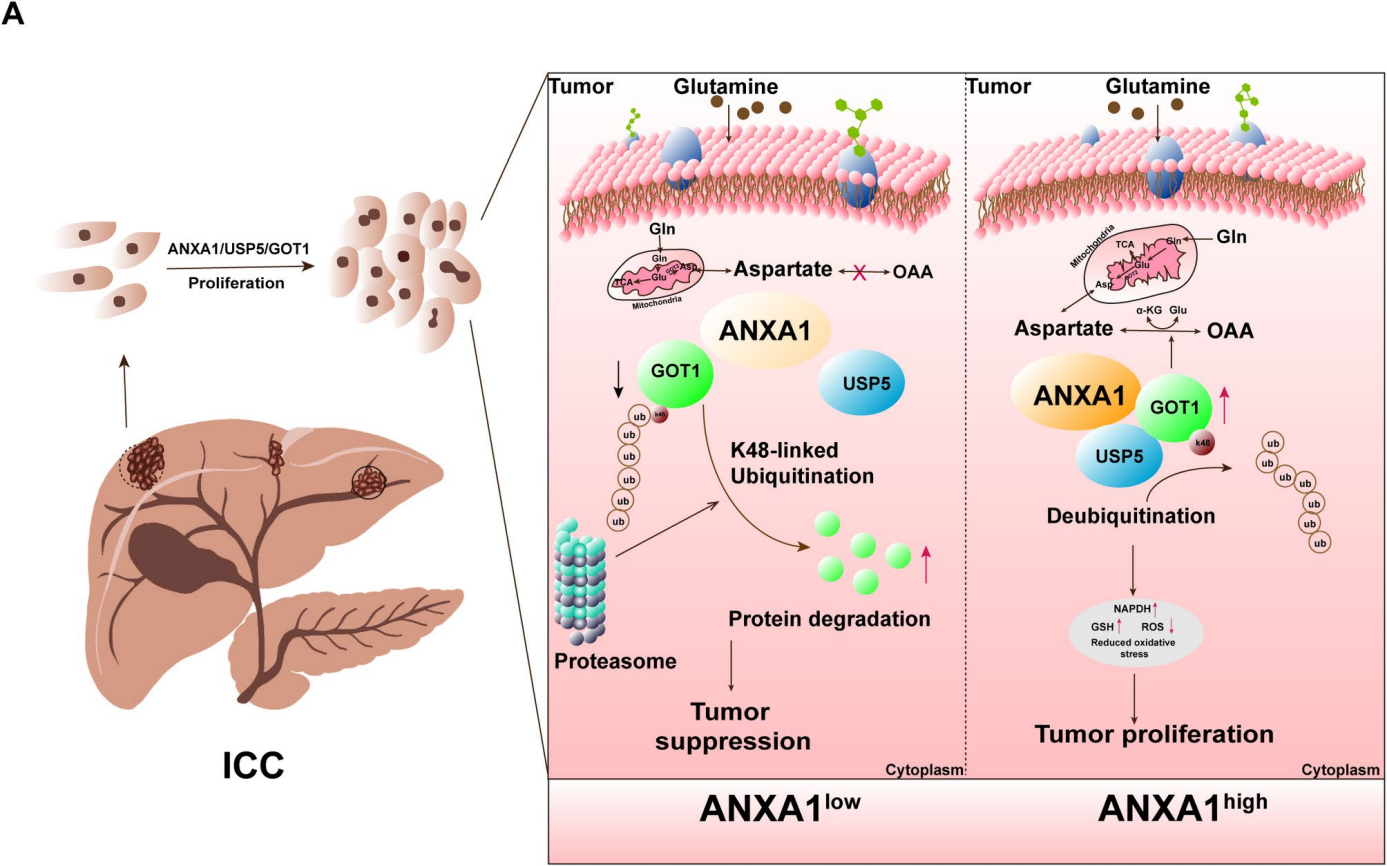
Summary illustration. **A**: Molecular mechanism diagram of ANXA1-USP5-GOT1 axis mediated glutamine metabolism promoting tumor growth and proliferation

In this study, we confirmed the stabilizing effect of ANXA1 on GOT1 protein and its promotion of ICC tumorigenesis. ANXA1, as a scaffold protein, highly expressed ANXA1 can recruit USP5 to stabilize GOT1, thereby promoting GOT1 mediated glutamine metabolism, further promoting GSH production and reducing ROS levels, reducing oxidative stress, and promoting ICC cell growth in vitro and in vivo (Fig. 8A). Therefore, our study revealed that the ANXA1-USP5-GOT1 axis is a key mechanism for regulating glutamine metabolism in ICC. Metabolic reprogramming is a hallmark of cancer, enabling tumor cells to meet the increased energy demands and improve the oxidative stress adaptation ability of tumor cells required for rapid proliferation, invasion, and metastasis [10, 32]. In addition to the metabolic imbalances driven by alterations in key metabolic enzyme genes, metabolism-related oncogenes significantly contribute to the occurrence and progression of ICC [33]. Identifying and characterizing key regulatory factors in important metabolic pathways can aid in uncovering new therapeutic targets and enhancing the selectivity of anticancer drugs. Through a multicenter, large-sample bioinformatic analysis of ICC, we identified key metabolic regulatory genes associated with tumor malignancy. Our findings revealed that amino acid metabolism was upregulated in ICC, with ANXA1 emerging as a key regulator of its occurrence and progression. ANXA1 was found to be significantly upregulated in tumor tissues and cells, and both in vitro and in vivo experiments confirmed its close association with ICC development. Accumulating evidence underscores the role of ANXA1 in tumorigenesis [34]. Furthermore, ANXA1 forms heterotetrameric complexes with S100A11, facilitating the formation of contact sites between the endoplasmic reticulum (ER) and endosomal membranes [28]. These complexes stabilize protein-protein interactions by serving as scaffold proteins. Our study demonstrated that ANXA1 acts as a scaffold bridging the interaction between the deubiquitinases USP5 and the GOT1. This interaction stabilized GOT1 and regulated glutamine metabolism, thereby promoting tumor proliferation. In the metabolic process of tumors, the function and abundance of key enzymes in metabolic pathways determined the metabolic types and levels of different tumors [35]. GOT1 is a cytoplasmic isoenzyme of glutamate oxaloacetate transaminase. Multiple studies demonstrated that GOT1 plays a pivotal role in regulating cell proliferation by participating in amino acid metabolism, particularly glutamine metabolism. Moreover, GOT1 is overexpressed in many cancers, and its association with the occurrence and progression of numerous tumors has been reported. For instance, inhibiting GOT1 was shown to induce ferroptosis-mediated death in pancreatic cancer cells [20]. GOT1 was crucial for the metabolic adaptation of cancer cells, particularly in those harboring KRAS mutations, where it coordinated glycolysis and oxidative phosphorylation pathways to support cell proliferation [36, 37]. Our research highlighted GOT1 as a key enzyme regulating glutamine metabolism in ICC. In this study, we revealed the critical role of the ANXA1–GOT1 axis in regulating glutamine metabolism and promoting ICC cell proliferation. This metabolic reprogramming may also further enhance tumor progression by modulating tumor-stroma interactions and immune responses. Research suggests that ANXA1 not only supports tumor growth through its effect on tumor cell metabolism but may also influence the immune microenvironment by regulating immune cell metabolism. ANXA1 regulates macrophage polarization by binding to formyl peptide receptors (FPRs), promoting the formation of M2 macrophages, which suppress anti-tumor immune responses [38]. Additionally, ANXA1 modulates the expression of cytokines such as IL-6 and VEGF, thereby influencing the inflammatory microenvironment of tumors [39, 40]. On the other hand, studies have shown that tumor cells competitively consume glutamine, restricting the glutamine metabolic pathway in T cells. This limitation impacts T cell activation, promotes M2 macrophage formation, and supports tumor proliferation and immune tolerance [41–43]. These findings suggest that the ANXA1–GOT1 axis may not only exert its function within tumor cells but also affect immune cells in the tumor microenvironment, further promoting tumor initiation and progression. The stabilization and degradation of key metabolic enzymes were closely linked to tumor progression. Regulation of ubiquitination and deubiquitination has garnered increasing attention for its role in cancer cell metabolic reprogramming [44, 45]. Abnormal transcriptional, translational, and post-translational regulation in cancer cells also affects ubiquitination and deubiquitination, exerting carcinogenic or tumor-suppressive effects [46]. Deubiquitinating enzymes were particularly significant in stabilizing oncogenes and driving tumor development. Ubiquitin-specific protease 5 (USP5) is an important deubiquitinating enzyme that regulates various physiological functions by removing ubiquitin chains from target proteins and plays a pivotal role in the occurrence and development of various cancers [25]. However, it remains unknown whether GOT1 harbors other binding partners and how these potential binding partners interact with USP5 and GOT1. Further experiments indicated that USP5 deubiquitinates and stabilizes GOT1 and that its stabilizing effect is regulated by ANXA1. This validated USP5 as a specific deubiquitinating enzyme for GOT1, thereby expanding the substrate pool of USP5.

In this study, we discovered that ANXA1 functions as a scaffold, connecting USP5 with GOT1 and facilitating their interaction. This scaffolding activity promotes GOT1 deubiquitination and stabilization, inhibiting its degradation via the ubiquitin-proteasome pathway. ANXA1 is highly expressed in patients with ICC, where it promotes tumor proliferation. MS analysis identified GOT1 and USP5 as binding partners of ANXA1. Mechanistically, ANXA1 facilitates the binding of USP5 to GOT1, enabling the removal of ubiquitin from K48 of GOT1, thereby preventing its proteasomal degradation. Notably, ANXA1 knockdown in combination with glutamine deprivation, significantly suppressed glutamine-mediated ICC cell proliferation. This study is the first to identify ANXA1 as a scaffold protein in ICC. Recent study indicates that some compounds based on molecular docking or high-throughput screening have the potential to interfere with the formation of specific protein complexes. Currently, small molecule inhibitors have made certain progress in disrupting protein-protein interactions. For instance, small molecules targeting the MDM2-p53 interaction, such as AMG 232, have entered clinical trials by restoring the tumor-suppressor function of p53 [47, 48]. PROTACs hijack E3 ligases and the ubiquitin-proteasome system (UPS), leading to selective degradation of the target proteins [49]. In our current research, we have found that ANXA1–USP5–GOT1 (58–129) plays a key role in this process. By using small molecule inhibitors to disrupt these key domains, it may play an important role in improving the progression of cholangiocarcinoma, providing new evidence for the development of new therapeutic targets. In future studies, we will further validate the therapeutic significance of these small molecules for intrahepatic cholangiocarcinoma through molecular docking or high-throughput screening. A promising finding from recent research: MDX-124, a humanized IgG1 monoclonal antibody that specifically targets annexin-A1 (ANXA1). MDX-124 has been shown to exert potent anti-tumor activity across a broad range of ANXA1-expressing cancer types, including pancreatic and triple-negative breast cancers, both in vitro and in vivo. Notably, MDX-124 induces G1-phase cell cycle arrest without triggering apoptosis and significantly reduces tumor growth [26]. Furthermore, MDX-124 has also demonstrated synergy with standard chemotherapy and immune checkpoint blockade [50], as shown in preclinical triple-negative breast cancer models.​Importantly, a first-in-human clinical trial of MDX-124 is currently underway to evaluate its safety and therapeutic efficacy in patients with advanced solid tumors known to overexpress ANXA1 [51]. These findings reinforce the translational potential of ANXA1-targeted therapy and support our current work by providing a promising therapeutic rationale for disrupting the ANXA1 axis in intrahepatic cholangiocarcinoma.

More importantly, we determined the impact of ANXA1 on GOT1-mediated glutamine metabolism and gained critical insights into the pathogenesis mechanisms underlying ICC, providing a basis for potential therapeutic targets.

## Conclusion

In summary, our study provides valuable insights into the use of ANXA1 as a scaffold protein to promote tumor occurrence and development through metabolic adaptation and confirms the tumor suppressive effect of ANXA1 inhibition combined with glutamine deprivation in vitro and in vivo, which has important therapeutic potential for intrahepatic cholangiocarcinoma.

## Electronic supplementary material

Below is the link to the electronic supplementary material.

Supplementary Material 1: Table 1: The detailed clinical information of ICC in this study.

Supplementary Material 2: Table 3: The primer sequences for qRT-PCR and siRNA target sequences, the plasmid sequences for cells construction and the primary antibodies used in the study.

Supplementary Material 3: Table 2: The raw datas of MS in this study.

Supplementary Material 4: Figures (S1-S7)..

## Data Availability

No datasets were generated or analysed during the current study.

## Declarations

relationships that could have appeared to influence the work reported in this paper.

## Abbreviations

ICC: Intrahepatic cholangiocarcinoma
ANXA1: Annexin A1
USP5: Ubiquitin Specific Protease 5
GOT1: Glutamate-oxaloacetate transaminase 1
TCGA: The Cancer Genome Atlas
GEO: Gene Expression Omnibus
NODE: China National Omics Data Encyclopedia
shRNA: Short hairpin RNA
GSEA: Gene Set Enrichment Analysis
CQ: Chloroquine
CHX: Cycloheximide
IHC: Immunohistochemistry
IP: Immunoprecipitation
BIOGRID: Biological General Repository for Interaction Datasets
CCLE: Cancer Cell Line Encyclopedia
