# Supplementary material for: ANXA1 promotes intrahepatic cholangiocarcinoma proliferation and growth by regulating glutamine metabolism through GOT1 stabilization
Source: J Exp Clin Cancer Res. 2025 May 19;44:151. doi: 10.1186/s13046-025-03400-z (PMC12087091; doi:10.1186/s13046-025-03400-z)
Supplement: Supplementary file 2 — Supplementary Material 2: Table 3: The primer sequences for qRT-PCR and siRNA target sequences, the plasmid sequences for cells construction and the primary antibodies used in the study. [file 13046_2025_3400_MOESM2_ESM.docx]

**Plasmid**

| Plasmid name | sequence(5'-3') | Cloning Vector | Vector element sequence |
| --- | --- | --- | --- |
| HA-Ubiquitin-K48 | CCCCTTGGCTTCGTTAGAACGCGGCTACAATTAATACATAACCTTATGTATCATACACATACGATTTAGGTGACACTATAGAATAACATCCACTTTGCCTTTCTCTCCACAGGTGTCCACTCCCAGGTCCAACTGCACCTCGGTTCTGCTAGCGTTTAAACGGGCCCTCTAGACGCCACCATGGGCTACCCCTATGATGTGCCTGACTACGCAGATCTCAATGGTGGTGGTGGTGGGTCGACCATGCAGATCTTCGTCAGAACGTTAACCGGTAGAACCATAACTCTAGAAGTTGAACCATCCGATACCATCGAAAACGTTAGAGCTAGAATTCAAGACAGAGAAGGCATTCCACCTGATCAACAAAGATTGATCTTTGCCGGTAAGCAGCTCGAGGACGGTAGAACGCTGTCTGATTACAACATTCAGAGAGAGTCCACCCTGCACCTGGTCCTCCGTCTCAGAGGTGGTTGATCTAGATAGTTAAACCGCTGATCAGCCTCGACTGTGCCTTCTAGTTGCCAGCCATCTGTTGTTTGCCCCTCCCCCGTGCCTTCCTTGACCCTGGAAGGTGCCACTCCCACTGTCCTTTCCTAATAAAATGAGGAAATTGCATCGCATTGTCTGAGTAGGTGTCATTCTATTCTGGGGGGTGGGGTGGGGCAGGACAGCAAGGGGGAGGATTGGGAAGAC | GV712 | CMV enhancer-MCS-SV40-puromycin |
| HA-Ubiquitin-K63 | ATAACCTTATGTATCATACACATACGATTTAGGTGACACTATAGAATAACATCCACTTTGCCTTTCTCTCCACAGGTGTCCACTCCCAGGTCCAACTGCACCTCGGTTCTGCTAGCGTTTAAACGGGCCCTCTAGACGCCACCATGGGCTACCCCTATGATGTGCCTGACTACGCAGATCTCAATGGTGGTGGTGGTGGGTCGACCATGCAGATCTTCGTCAGAACGTTAACCGGTAGAACCATAACTCTAGAAGTTGAACCATCCGATACCATCGAAAACGTTAGAGCTAGAATTCAAGACAGAGAAGGCATTCCACCTGATCAACAAAGATTGATCTTTGCCGGTAGACAGCTCGAGGACGGTAGAACGCTGTCTGATTACAACATTCAGAAGGAGTCCACCCTGCACCTGGTCCTCCGTCTCAGAGGTGGTTGATCTAGATAGTTAAACCGCTGATCAGCCTCGACTGTGCCTTCTAGTTGCCAGCCATCTGTTGTTTGCCCCTCCCCCGTGCCTTCCTTGACCCTGGAAGGTGCCACTCCCACTGTCCTTTCCTAATAAAATGAGGAAATTGCATCGCATTGTCTGAGTAGGTGTCA | GV712 | CMV enhancer-MCS-SV40-puromycin |
| HA-Ubiquitin-WT | ACCCCCTTGGCTTCGTTAGAACGCGGCTACAATTAATACATAACCTTATGTATCATACACATACGATTTAGGTGACACTATAGAATAACATCCACTTTGCCTTTCTCTCCACAGGTGTCCACTCCCAGGTCCAACTGCACCTCGGTTCTGCTAGCGTTTAAACGGGCCCTCTAGACGCCACCATGGGCTACCCCTATGATGTGCCTGACTACGCAGATCTCAATGGTGGTGGTGGTGGGTCGACCATGCAGATCTTCGTGAAGACCCTGACTGGTAAGACCATCACTCTCGAAGTGGAGCCGAGTGACACCATTGAGAATGTCAAGGCAAAGATCCAAGACAAGGAAGGCATCCCTCCTGACCAGCAGAGGTTGATCTTTGCTGGGAAACAGCTGGAAGATGGACGCACCCTGTCTGACTACAACATCCAGAAAGAGTCCACCCTGCACCTGGTCCTCCGTCTCAGAGGTGGTTGATCTAGATAGTTAAACCGCTGATCAGCCTCGACTGTGCCTTCTAGTTGCCAGCCATCTGTTGTTTGCCCCTCCCCCGTGCCTTCCTTGACCCTGGAAGGTGCCACTCCCACTGTCCTTTCCTAATAAAATGAGGAAATTGCATCGCATTGTCTGAGTAGGTGTCATTCTATTCTGGGGGGTGGGGTGGGGCAGGACAGCAAGGGGGAGGATTGGGAAGACAATAGCAGG | GV712 | CMV enhancer-MCS-SV40-puromycin |
| Flag-ANXA1 | ATGGCAATGGTATCAGAATTCCTCAAGCAGGCCCGTTTTCTTGAAAATCAAGAACAGGAATATGTTCAAGCTGTAAAATCATACAAAGGTGGTCCTGGGTCAGCAGTGAGCCCCTACCCTTCCTTCAATGTATCCTCGGATGTTGCTGCCTTGCACAAAGCTATCATGGTTAAAGGTGTGGATGAAGCAACCATCATTGACATTCTTACCAAGAGGACCAATGCTCAGCGCCAGCAGATCAAGGCCGCGTACTTACAGGAGAATGGAAAGCCCTTGGATGAAGTCTTGAGAAAAGCCCTTACAGGCCACCTGGAGGAGGTTGTTTTGGCTATGCTAAAAACTCCAGCTCAGTTTGATGCAGATGAACTCCGTGGTGCCATGAAGGGACTTGGAACAGATGAAGACACTCTCATTGAGATTTTGACAACAAGATCTAACGAACAAATCAGAGAGATTAATAGAGTCTACAGAGAAGAGCTGAAAAGAGATCTGGCCAAAGACATCACTTCAGATACATCTGGAGACTTTCGGAAAGCCTTGCTTGCTCTTGCCAAGGGTGACCGTTGTCAGGACTTGAGTGTGAATCAAGATTTGGCTGATACAGATGCCAGGGCTTTGTATGAAGCTGGAGAAAGGAGAAAGGGGACAGACGTGAACGTCTTCACCACAATTCTGACCAGCAGGAGCTTTCCTCATCTTCGCAGAGTGTTTCAGAATTACGGAAAGTACAGTCAACATGACATGAACAAAGCTCTGGATCTGGAACTGAAGGGTGACATTGAGAAGTGCCTCACAACCATCGTGAAGTGTGCCACCAGCACTCCAGCTTTCTTTGCCGAGAAGCTGTACGAAGCCATGAAGGGTGCCGGAACTCGCCATAAGGCATTGATCAGGATTATGGTCTCCCGTTCGGAAATTGACATGAATGAAATCAAAGTATTTTACCAGAAGAAGTATGGAATCTCTCTTTGCCAAGCCATCCTGGATGAAACCAAAGGAGACTATGAAAAAATCCTGGTGGCTCTGTGTGGTGGAAACGATTACAAGGATGACGACGATAAGTAG | GV492 | Ubi-MCS-3FLAG-CBh-gcGFP-IRES-puromycin |
| Sh-ANXA1#1 | Target sequence: GCATTCTATCAGAAGATGTAT | GV493 | hU6-MCS-CBh-gcGFP-IRES-puromycin |
| Sh-ANXA1#2 | Target sequence:GCCTTGTATGAAGCAGGAGAA | GV493 | hU6-MCS-CBh-gcGFP-IRES-puromycin |
| Myc-USP5 | GACAGACGTGAACGTCTTCACCACAATTCTGACCAGCAGGAGCTTTCCTCATCTTCGCAGAGTGTTTCAGAATTACGGAAAGTACAGTCAACATGACATGAACAAAGCTCTGGATCTGGAACTGAAGGGTGACATTGAGAAGTGCCTCACAACCATCGTGAAGTGTGCCACCAGCACTCCAGCTTTCTTTGCCGAGAAGCTGTACGAAGCCATGAAGGGTGCCGGAACTCGCCATAAGGCATTGATCAGGATTATGGTCTCCCGTTCGGAAATTGACATGAATGAAATCAAAGTATTTTACCAGAAGAAGTATGGAATCTCTCTTTGCCAAGCCATCCTGGATGAAACCAAAGGAGACTATGAAAAAATCCTGGTGGCTCTGTGTGGTGGAAACGATTACAAGGATGACGACGATAAGTAG | GV492 |  |
| Sh-USP5#2 | Target sequence: GATAGACATGAACCAGCGGAT | GV493 | hU6-MCS-CBh-IRES-puromycin |
| Sh-USP5#3 | Target sequence: GACCACACGATTTGCCTCATT | GV493 | hU6-MCS-CBh-IRES-puromycin |
| Flag-GOT1 | GCAATGGTATCAGAATTCCTCAAGCAGGCCCGTTTTCTTGAAAATCAAGAACAGGAATATGTTCAAGCTGTAAAATCATACAAAGGTGGTCCTGGGTCAGCAGTGAGCCCCTACCCTTCCTTCAATGTATCCTCGGATGTTGCTGCCTTGCACAAAGCTATCATGGTTAAAGGTGTGGATGAAGCAACCATCATTGACATTCTTACCAAGAGGACCAATGCTCAGCGCCAGCAGATCAAGGCCGCGTACTTACAGGAGAATGGAAAGCCCTTGGATGAAGTCTTGAGAAAAGCCCTTACAGGCCACCTGGAGGAGGTTGTTTTGGCTATGCTAAAAA | GV492 | Ubi-MCS-3FLAG-CBh-IRES-puromycin |
| Sh-GOT1#1 | Target sequence: CCAAGCCATCCTGGATGAAAC | GV493 | hU6-MCS-CBh-IRES-puromycin |
| Sh-GOT1#2 | Target sequence: GAAACGATTACAAGGATGACG | GV493 | hU6-MCS-CBh-IRES-puromycin |
| LV-NC | Primer locations and sequences：  Ubi-F（3756-3778）：GGGTCAATATGTAATTTTCAGTG  FLAG-R-2（3940-3919）：CCTTATAGTCCTTATCATCGTC | GV492 |  |
| Sh-NC | Primer locations and sequences：  pGCSIL-F（2637-2657）：CCATGATTCCTTCATATTTGC | GV493 |  |

**Small interfering RNA**

| siRNA name | sense（5'-3'） | antisense（5'-3'） |
| --- | --- | --- |
| Negative control | UUCUCCGAACGUGUCACGUTT | ACGUGACACGUUCGGAGAATT |
| SiRNA-ANXA1(1) | GCCUUGUAUGAAGCAGGAGAATT | UUCUCCUGCUUCAUACAAGGCTT |
| SiRNA-ANXA1(2) | GCAACCAUCAUUGACAUUCUATT | UAGAAUGUCAAUGAUGGUUGCTT |
| SiRNA-ANXA1(3) | GCAUUCUAUCAGAAGAUGUAUTT | AUACAUCUUCUGAUAGAAUGCTT |
|  |  |  |

**Primers used for PCR**

| Gene name | Forward | Reverse |
| --- | --- | --- |
| Human+ANXA1 | GAGCCCCTATCCTACCTTCAATC | GCTTCATCCACACCTTTAACCAT |
| Human+USP5 | GCTGCTGTCAGTATTACCGAC | AAAGCCCAGAAACGTGTTCATA |
| Human+GOT1 | GGAGCAGAAGATTGCTAATGACA | AAGGGCAAGACGAGAAGCAC |
| Human+α-Tubulin | CGTGTTCGGCCAGAGTGGTGC | GGGTGAGGGCATGACGCTGAA |

**Antibody and Reagents**

| Antibody Name | KDa | Dilution | Source |  |
| --- | --- | --- | --- | --- |
| Annexin A1 Polyclonal antibody | 37 | 1:5000 | Proteintech，Wuhan，CHINA | Cat No. 21990-1-AP |
| USP5 Polyclonal antibody | 96 | 1:5000 | Proteintech，Wuhan，CHINA | Cat No. 10473-1-AP |
| GOT1 Polyclonal antibody | 46 | 1:1500 | Proteintech，Wuhan，CHINA | Cat No. 14886-1-AP |
| DYKDDDDK tag Polyclonal antibody (Binds to FLAG® tag epitope) |  | 1:20000 | Proteintech，Wuhan，CHINA | Cat No. 20543-1-AP |
| MYC tag Polyclonal antibody |  | 1:5000 | Proteintech，Wuhan，CHINA | Cat No. 16286-1-AP |
| ubiquitin Polyclonal antibody |  | 1:5000 | Proteintech，Wuhan，CHINA | Cat No. 10201-2-AP |
| Reagents | Source | | |  |
| Glutamine Assay kit | Abcam，Cambridge，UK | | | ab197011 |
| Glutamate Assay kit | Abcam，Cambridge，UK | | | ab83389 |
| Aspartate (Asp) Colorimetric Assay Kit | Elabscience，Wuhan，CHINA | | | E-BC-K849-M |
| Reduced Glutathione (GSH) Colorimetric Assay Kit | Elabscience, Wuhan, China | | | E-BC-K030-M |
| Reactive Oxygen Species (ROS) Fluorometric Assay Kit (Green) | Elabscience, Wuhan, China | | | E-BC-K138-F |
