# Supplementary material for: ANXA1 promotes intrahepatic cholangiocarcinoma proliferation and growth by regulating glutamine metabolism through GOT1 stabilization
Source: J Exp Clin Cancer Res. 2025 May 19;44:151. doi: 10.1186/s13046-025-03400-z (PMC12087091; doi:10.1186/s13046-025-03400-z)
Supplement: Supplementary file 4 — Supplementary Material 4: Figures (S1-S7). [file 13046_2025_3400_MOESM4_ESM.pdf]

Supplementary Figures

Figure S1

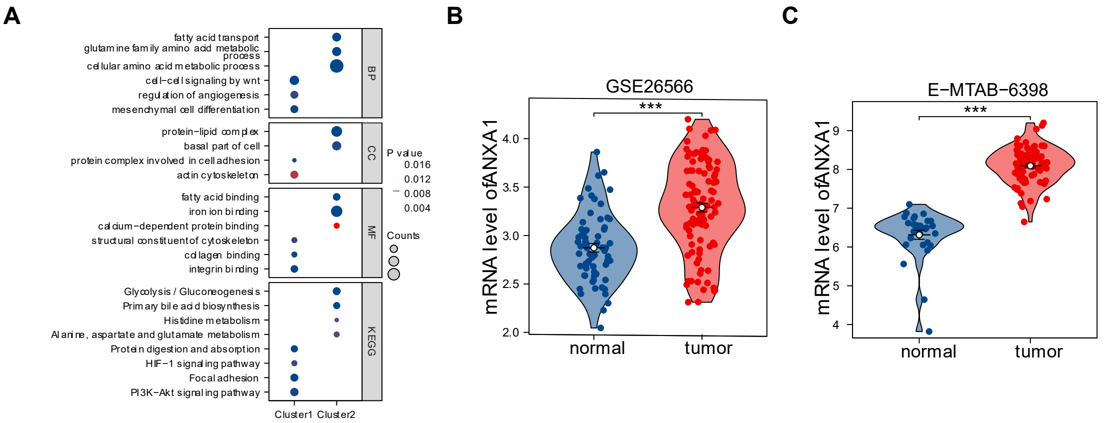

A: GO and KEGG enrichment analysis of genes with differences in two metabolic types based on biological analysis.

B-C: The expression levels of ANXA1 mRNA in the public databases GSE26566 and E-MTAB-6398. (Mann–Whitney U test, ***, p<0.001)

Figure S2

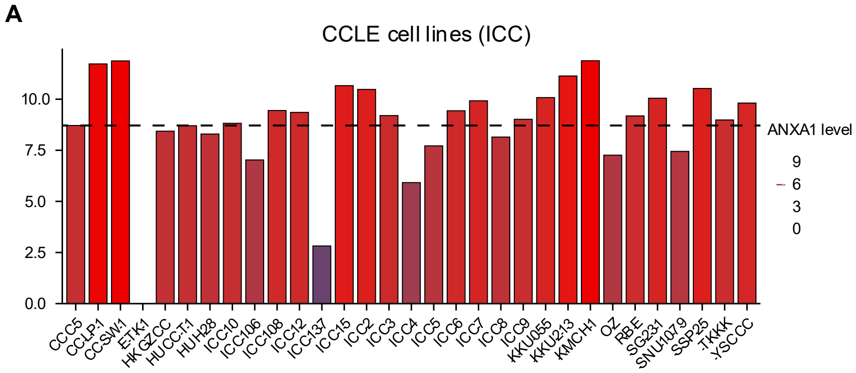

A: mRNA levels of ANXA1 in intrahepatic cholangiocarcinoma cell lines from CCLE.

Figure S3

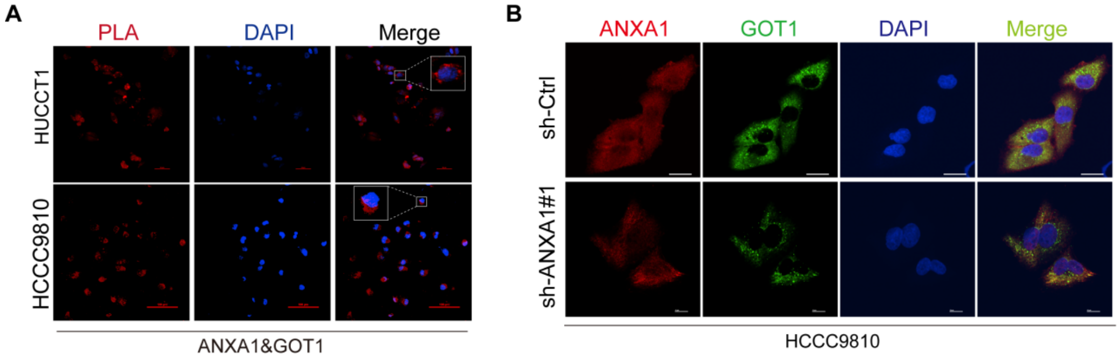

A： PLA signals (red puncta) indicate direct binding between ANXA1 and GOT1 in HUCCT1 and HCCC9810 cells, Nuclei were counterstained with DAPI (blue) (Scale bars, 50 μm/100μm).

B： Immunofluorescence co-localization of ANXA1 and GOT1 in HUCCT1 cells with or without knockdown of ANXA1(Scale bars, 10μm).

Figure S4

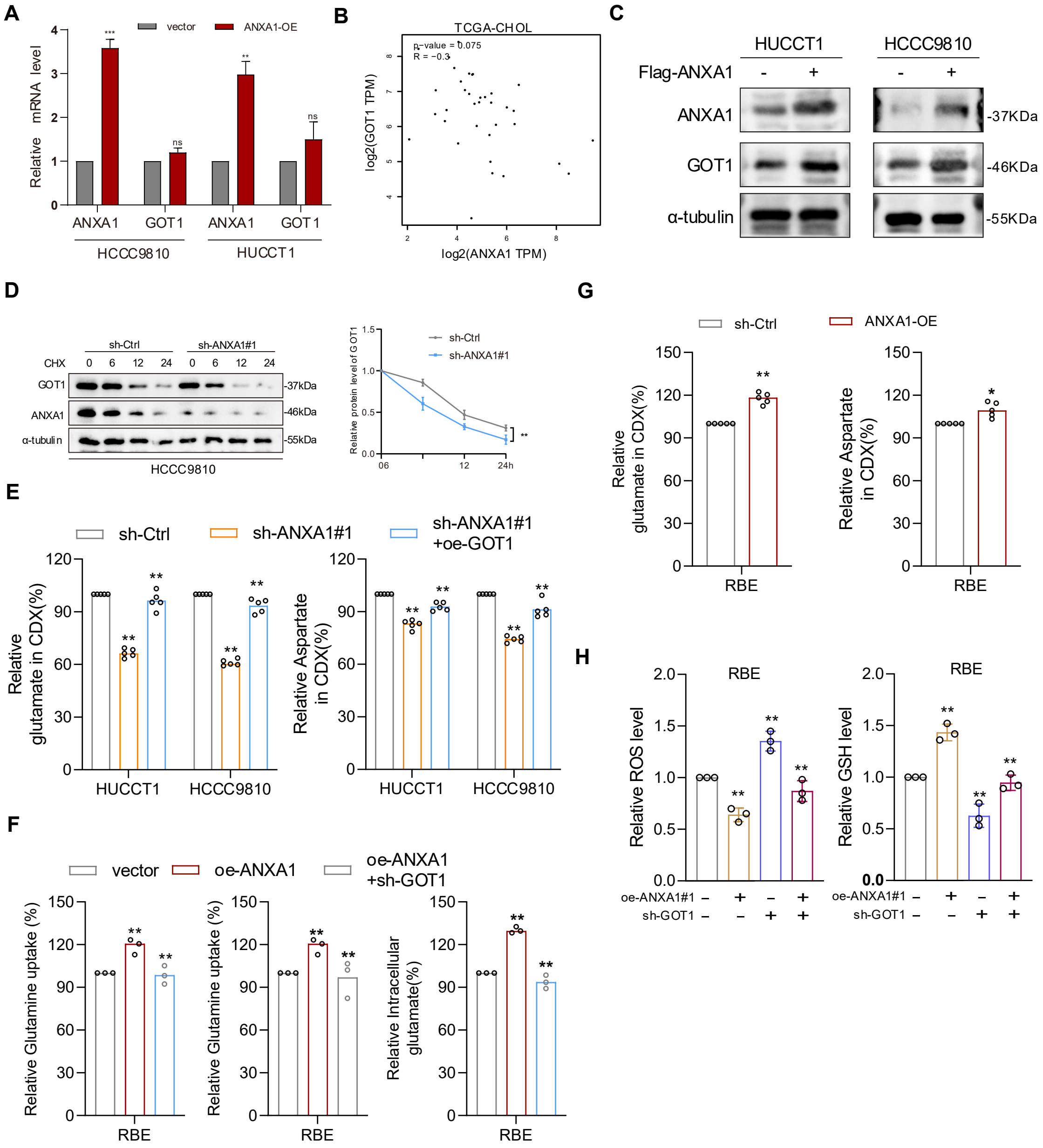

A: RT-PCR detecting the mRNA levels of GOT1 after overexpression of ANXA1 in HUCCT1 and HCCC9810 cells. (Mean values (n = 3) ± s.d. One-way ANOVA, **, p < 0.01; ns, no significance)

B: Analysis of mRNA expression levels of ANXA1 and GOT in GEPIA public database.

C: IB of GOT1, ANXA1, and α-Tubulin in HUCCT1 and HCCC9810 cells with ANXA1 overexpression.

D: Protein levels of GOT1 in HCCC9810 cells with or without USP5 knockdown after CHX treatment (100μg/ml) for the indicated times. (Mean values (n = 3) ± s.d; two-way ANOVA, **, p < 0.01)

E: Glutamate or aspartate levels were examined in CDX with stable knockdown ANXA1 or overexpress GOT1. (Mean values (n = 5) ± s.d; one-way ANOVA, **, p < 0.01)

F: Glutamine and glutamate or aspartate levels were examined in RBE with stable of overexpress ANXA1 or knockdown GOT1.(Mean values (n = 3) ± s.d; one-way ANOVA, **, p < 0.01.)

G: Glutamate or aspartate levels were examined in RBE with stable overexpress ANXA1.（Mean values (n = 5) ± s.d.one-way ANOVA, **, p < 0.01.）

H: ROS and GSH level of RBE detecting with ANXA1 overexpressed and or knockdown GOT1. (Mean values (n = 3) ± s.d.one-way ANOVA, **, p < 0.01.)

Figure S5

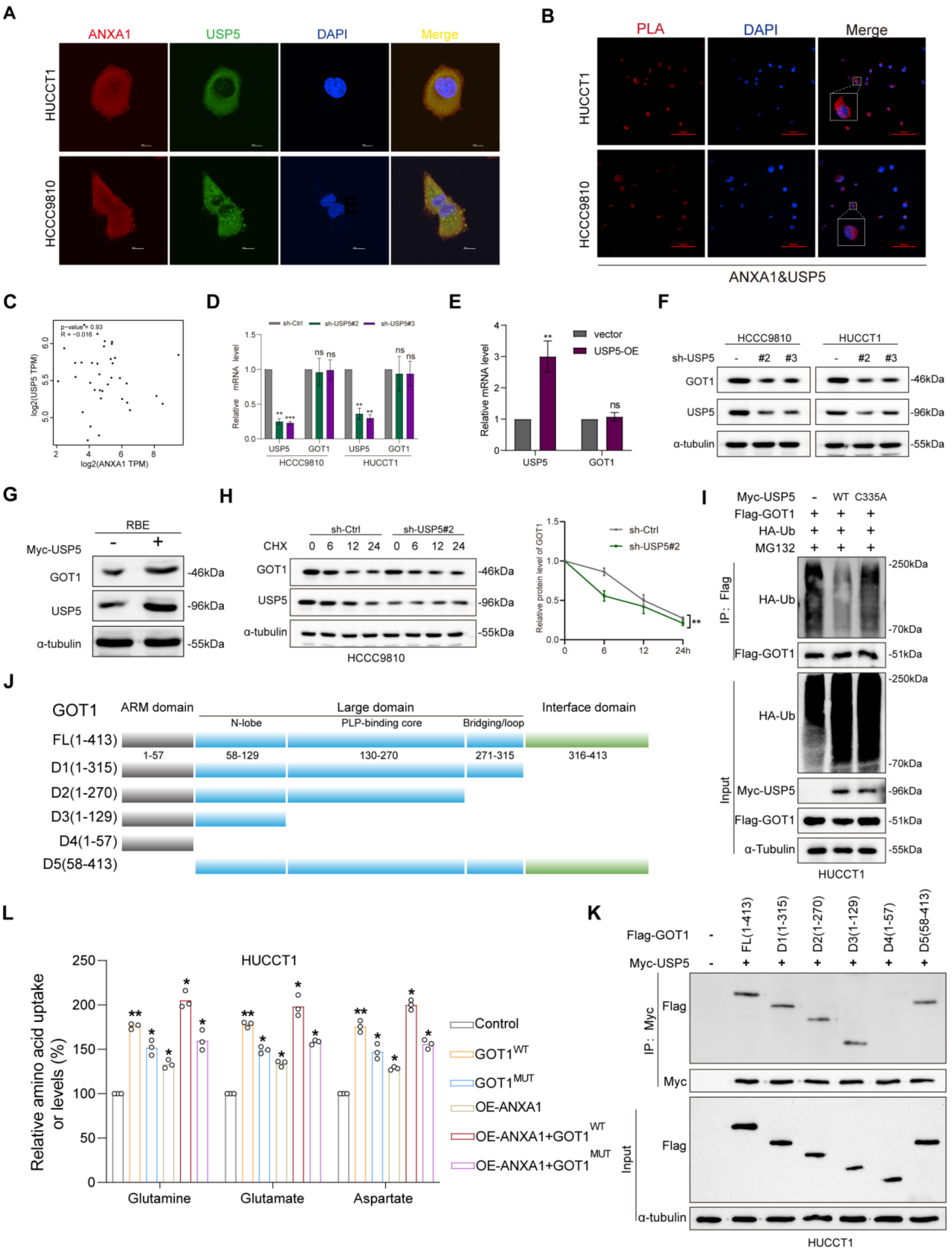

A： Immunofluorescence co-localization of ANXA1 and USP5 in HUCCT1 and HCCC9810 cells.

B：PLA signals (red puncta) indicate direct binding between ANXA1 and USP5 in HUCCT1 and HCCC9810 cells. Nuclei were counterstained with DAPI (blue) (Scale bars, 100 μm).

C: Analysis of mRNA expression levels of ANXA1 and USP5 in GEPIA public database.

D: RT-PCR detecting the mRNA levels of GOT1 after knockdown of USP5 in HUCCT1 and HCCC9810 cells. (Mean values (n = 3) ± s.d. Two-way ANOVA, **, $p < 0.01$; ns, no significance)

E: RT-PCR detecting the mRNA levels of GOT1 after overexpression of USP5 in RBE cells. (Mean values (n = 3) ± s.d. Two-way ANOVA, **, $p < 0.01$. ns means no significance)

F: IB of GOT1, USP5, and α-Tubulin in HUCCT1 and HCCC9810 cells with USP5 knockdown.

G: IB of GOT1, USP5, and α-Tubulin in RBE cells with USP5 overexpression.

H: Protein levels of GOT1 in HCCC9810 cells with or without USP5 knockdown after CHX treatment (100μg/ml) for the indicated times. (Mean values (n = 3) ± s.d. Two-way ANOVA, **, $p < 0.01$.)

I: HUCCT1 cells were transfected with Flag-GOT1, HA-tagged ubiquitin (HA-Ub), and either wild-type (WT) or catalytically inactive mutant (C335A) Myc-tagged USP5, followed by treatment with MG132 (10 μM, 6 h). Cell lysates were subjected to immunoprecipitation (IP) using anti-Flag antibody and probed with anti-HA and anti-Flag antibodies to detect ubiquitinated GOT1 and total GOT1 levels.

J: Schematic diagram of truncated GOT1 constructs (D1–D5) used to map the USP5-binding region. The full-length GOT1 (FL, 1–413 aa) contains an ARM domain (1–57), a large domain including the N-lobe (58–129), PLP-binding core (130–270), bridging loop (271–315), and an interface domain (316–413).

K: Co-immunoprecipitation of Myc-USP5 with Flag-tagged full-length or truncated GOT1 constructs in HUCCT1 cells.

L: Quantification of intracellular amino acid levels (glutamine uptake and metabolic derivatives) in HUCCT1 cells transfected with $GOT1^{WT}$ or $GOT1^{MUT}$, with or without ANXA1 overexpression. (Mean values (n = 3) ± s.d. One-way ANOVA ,*,$p < 0.05$; **,$p < 0.01$.)

Figure S6

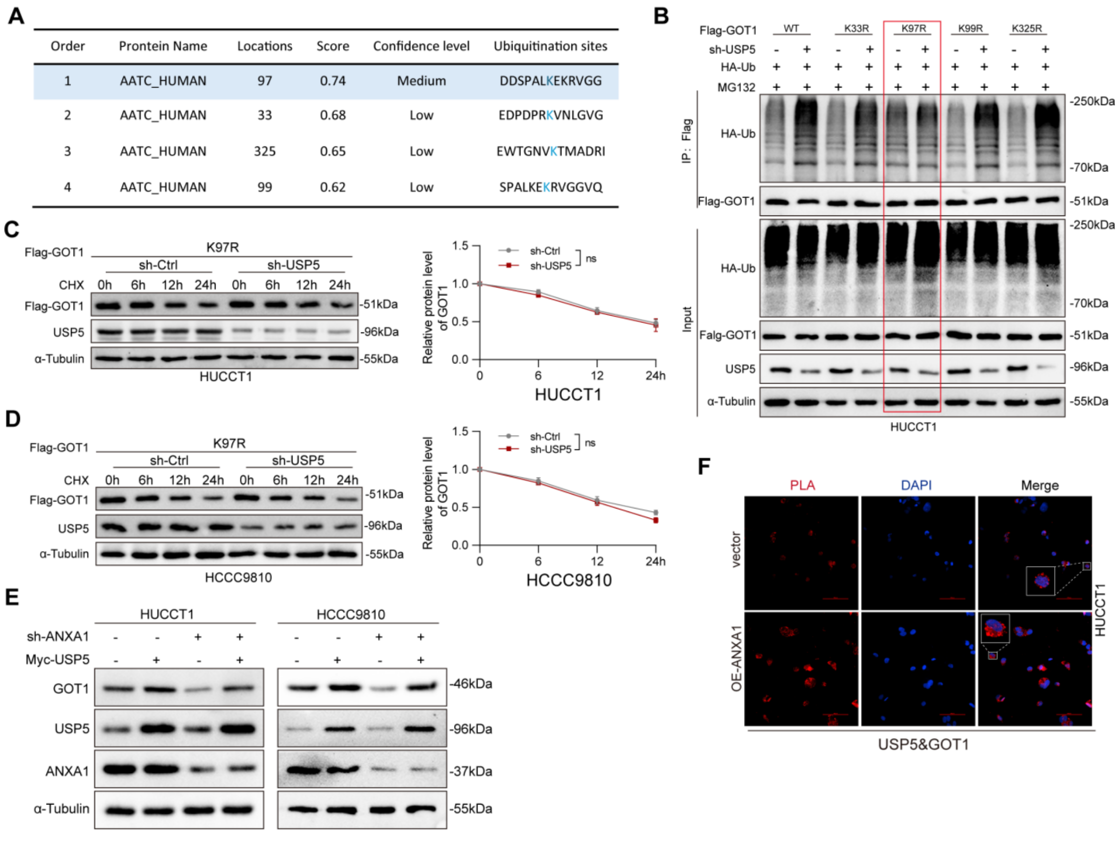

A: Used the MATLAB Compiler Runtime (MCR, version: R2009a MCR 7.10) tool to analyze all lysine residues in the GOT1 amino acid sequence. Lysines with a prediction score ≥0.62 were considered likely to be ubiquitinated.

B： Site-directed lysine mutants of GOT1 (K33R, K97R, K99R, K325R) or wild-type GOT1 (Flag-GOT1-WT) were transfected into HUCCT1 cells, with or without USP5 knockdown (sh-USP5), along with HA-Ub and MG132. Flag-GOT1 was immunoprecipitated and probed for HA-Ub to assess GOT1 ubiquitination.

C-D： In HUCCT1 and HCCC9810 cells, Flag-tagged GOT1 K97R mutant constructs were transfected with or without USP5 knockdown. Cells were then treated with cycloheximide (CHX, 100 μg/mL) to inhibit protein synthesis, and samples were collected at 0, 6, 12, and 24 hours. GOT1 protein levels were assessed by immunoblotting using an anti-Flag antibody, and protein stability was quantified. (mean value(n=3) ± sd. Two-way ANOVA; ns, no significance.)

E： Western blot analysis of GOT1, USP5, and ANXA1 protein expression in HUCCT1 and HCCC9810 cells transfected with sh-ANXA1 and/or Myc-USP5 plasmids.

F： PLA signals for USP5–GOT1 interaction in control (top) and ANXA1-overexpressing (OE-ANXA1, bottom) cells (Scale bars, 100 μm).

Figure S7

A

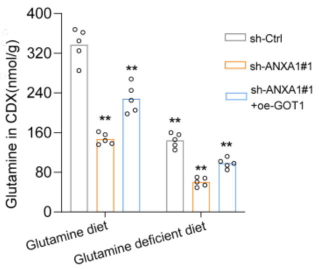

A：Glutamine concentrations were measured in subcutaneous xenograft tumors (CDX) derived from HUCCT1 cells subjected to different treatments: sh-Ctrl, sh-ANXA1#1, and sh-ANXA1#1+oe-GOT1. (mean ± s.d (n = 5). **, $p < 0.01$; One-way ANOVA
